# Transcriptome Analysis of the Desert Locust Central Nervous System: Production and Annotation of a *Schistocerca gregaria* EST Database

**DOI:** 10.1371/journal.pone.0017274

**Published:** 2011-03-21

**Authors:** Liesbeth Badisco, Jurgen Huybrechts, Gert Simonet, Heleen Verlinden, Elisabeth Marchal, Roger Huybrechts, Liliane Schoofs, Arnold De Loof, Jozef Vanden Broeck

**Affiliations:** Department of Animal Physiology and Neurobiology, Katholieke Universiteit Leuven, Leuven, Belgium; University of Nebraska Medical Center, United States of America

## Abstract

**Background:**

The desert locust (*Schistocerca gregaria*) displays a fascinating type of phenotypic plasticity, designated as ‘phase polyphenism’. Depending on environmental conditions, one genome can be translated into two highly divergent phenotypes, termed the solitarious and gregarious (swarming) phase. Although many of the underlying molecular events remain elusive, the central nervous system (CNS) is expected to play a crucial role in the phase transition process. Locusts have also proven to be interesting model organisms in a physiological and neurobiological research context. However, molecular studies in locusts are hampered by the fact that genome/transcriptome sequence information available for this branch of insects is still limited.

**Methodology:**

We have generated 34,672 raw expressed sequence tags (EST) from the CNS of desert locusts in both phases. These ESTs were assembled in 12,709 unique transcript sequences and nearly 4,000 sequences were functionally annotated. Moreover, the obtained *S. gregaria* EST information is highly complementary to the existing orthopteran transcriptomic data. Since many novel transcripts encode neuronal signaling and signal transduction components, this paper includes an overview of these sequences. Furthermore, several transcripts being differentially represented in solitarious and gregarious locusts were retrieved from this EST database. The findings highlight the involvement of the CNS in the phase transition process and indicate that this novel annotated database may also add to the emerging knowledge of concomitant neuronal signaling and neuroplasticity events.

**Conclusions:**

In summary, we met the need for novel sequence data from desert locust CNS. To our knowledge, we hereby also present the first insect EST database that is derived from the complete CNS. The obtained *S. gregaria* EST data constitute an important new source of information that will be instrumental in further unraveling the molecular principles of phase polyphenism, in further establishing locusts as valuable research model organisms and in molecular evolutionary and comparative entomology.

## Introduction

For many decades, locusts have proven to be important model organisms for insect physiological research, in particular for the study of endocrinological and neurobiological processes. Their relatively large size has enabled the purification and identification of an extensive repertoire of nearly a hundred biologically active regulatory peptides [1]–[5]. Unlike many other insect model species, such as the fruit fly, the honey bee and the silk worm, locusts belong to the hemimetabolous branch of insects. This subgroup comprises insects that undergo an incomplete metamorphosis, lacking the formation of a pupal stage. The rapidly expanding genome and transcriptome data have given research in several holometabolous model insects an unprecedented impetus. Although genome data have recently become available for the hemimetabolous species *Acyrthosiphon pisum* (pea aphid) [6] and *Pediculus humanus* (human body louse) [7], sequence information from this branch of insects is still lagging behind. In addition, locusts appear to have a very big genome (estimated 2–3 times larger than the human genome [8]), which constitutes a major hurdle to completely sequence it.

In addition, the desert locust, *Schistocerca gregaria*, is particularly well known as a notorious swarm-forming insect species, which can inflict devastating damage to the agricultural production in large areas of the world (*cf.* FAO website: http://www.fao.org/ag/locusts/). Intriguingly, the same species can also occur in a more harmless solitarious form, which tends to avoid the company of other locusts. Besides this very prominent behavioral distinction, solitarious and gregarious locusts also differ in many other traits such as coloration, morphology, developmental and reproductive physiology. The existence of these two extremely different forms or phases, also designated as phase polyphenism, is a fascinating example of phenotypic plasticity, whereby two obviously different phenotypes are encoded by the same genome [9]–[11]. [In laboratory conditions the two locust phases are referred to as isolated-reared (solitarious) and crowded-reared (gregarious).] Conversion between the two phases is termed phase transition, which is a reversible, continuous process that is accompanied by the occurrence of several intermediate forms [9], [10]. Development towards the gregarious phase is triggered by an increase in population density. Remarkably, a behavioral shift can be observed from mutual aversion to aggregation within a few hours of crowding [12]–[14].

The central nervous system (CNS) plays a crucial role in these early gregarization effects. Sensory stimuli generated by the presence of other locusts can induce changes in the titers of several neurotransmitters [14]–[16]. The involvement of the CNS is not surprising since it constitutes the primary systemic control center that is integrating sensory input, generates behavioral responses and regulates many physiological processes. In addition, although crowded-reared locusts are on average smaller, their brain was found to be 30% larger than that of isolated-reared animals and to be differently proportionated [17]. Elevated population density leads to increased competition for food and forces the locusts to alter their foraging strategy. Since foraging behavior and social life style have already been associated with differences in the brain volume of insects [18]–[23], these may also be involved in distinguishing gregarious from solitarious brain size [17]. Furthermore, serotonin has been demonstrated to be a crucial central mediator of the behavioral phase transformation [13]. During the first hours of forced crowding a temporary increase in serotonin has been observed in the thoracic ganglia [15]. However, development towards the gregarious phase is not only characterized by a behavioral shift. In later stages of gregarization (which can comprise several generations) multiple physiological processes are affected. These include reproduction, development and determination of life span [11], [24]. However, to a great extent, the molecular basis underlying all these phenotypic changes still remains elusive. Therefore, additional sequence information (both nucleotide and protein sequence information) is currently needed to allow further investigations of the mechanisms underlying phase-dependent physiological processes in locusts.

In order to compensate for the absence of locust genome data (and for the difficulty to obtain such data, given the huge estimated size of their genome), we have currently produced an EST (‘Expressed Sequence Tags’) database representing transcripts expressed in the CNS of the desert locust, *S. gregaria*. Furthermore, to our knowledge, we hereby present the first EST database derived exclusively from a complete insect CNS (head ganglia and ventral nerve cord).

An EST database (LocustDB) derived from various body parts was already publicly available for a related locust species, *Locusta migratoria* [25], [26]. Within the family of Acrididae *S. gregaria* and *L. migratoria* belong to the subfamilies of Cyrtacanthacridinae and Oedipodinae, respectively. Not all genera or species classified under both subfamilies display phase polyphenism, indicating that the desert locust and the migratory locust must have developed this phenotypic plasticity independently and/or that other species may have lost this ability. Most probably, the truth lies somewhere in between. Song suggested that phase polyphenism is a very complex character that results from interactions between several density-dependent plastic traits, which may have evolved differently. Behavioral phenotypic traits seem to be less well conserved than physiological traits, which may explain why not all grasshopper species within the subfamilies of Cyrtacanthacridinae or Oedipodinae display phase polyphenism [27]–[29]. In addition, the neural mechanisms inducing the behavioral switch may differ among different locust species. Visual and olfactory incentives and tactile stimulation of the hind legs act synergistically to trigger gregarization behavior in *S. gregaria* [30], [31]. However, it was recently demonstrated that these stimuli do probably not result in a behavioral change in the Australian plague locust (*Chortoicetes terminifera*), which, like *L. migratoria*, belongs to the subfamily of the Oedipodinae. In these animals, tactile stimulation of the antennae is suggested to be the primary gregarizing input [32]. This example emphasizes that phase polyphenism research in different locust species may have to be approached differently.

Therefore, by specifically focusing on the desert locust CNS, information in the obtained database is not only expected to complement previously obtained orthopteran transcript sequence data. In addition, the *S. gregaria* database will also be instrumental for a more detailed molecular dissection of neuronal and neuro-endocrine control mechanisms and signaling pathways responsible for many physiological processes, including the very fascinating process of locust phase transition. Information in this *S. gregaria* EST database is compared to that in other organisms' databases (both genome and EST). Annotation of the EST sequences and assignment of ‘Gene Ontology’ (GO) terms demonstrates that a broad range of GO categories is represented in this novel database. Since many transcripts encode products that are predicted to play a role in neuronal signaling or in signal transduction, this paper will focus on these particular sequences and provide an overview of their possible role(s). Furthermore, a selection of genes showing differential expression in isolated- and crowded-reared desert locusts is retrieved from the database.

## Results and Discussion

### 1. An EST database for *Schistocerca gregaria* CNS

#### Production of the EST database

**Figure 1:**
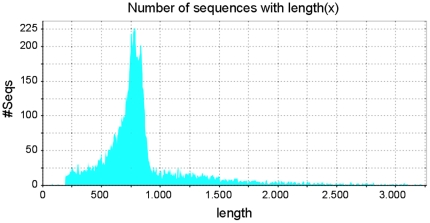
Sequence length distribution of the 12,709 unique *S. gregaria* EST sequences (4,785 contigs/7,924 singletons).

Normalized cDNA libraries were derived from microdissected CNS of isolated- and crowded-reared, larval and adult desert locust males and females. In total, 34,672 raw EST data have been generated from these libraries. These ESTs were further assembled in 4,785 contigs and 7,924 singletons, resulting in a total of 12,709 unique sequences. A sequence length distribution analysis (Figure 1) shows that for most of these uniques between 600–900 nucleotides of high quality information (≥99% confidence level) is available, although several longer sequences were also observed. Sequence data have been integrated in a desert locust ‘Expressed Sequence Tag Information Management and Annotation’ (ESTIMA) database (http://titan.biotec.uiuc.edu/locust/), which has several functionalities for retrieval of EST-related information. An overview of what these functionalities accept as query/input and what they produce as output is given in Table 1.

**Table 1:** Overview of the six main ESTIMA functionalities for retrieval of *S. gregaria* EST-related information.

Functionality	Input	Output
GO browser	Key word	GO tree
	GO term ID	EST count per term
	Root category	
Sequence ID	Sequence ID	Chromatogram
		Download raw/trimmed sequence
		Alignment length (only contigs)
		GenBank accession ID
		Contig structure (only contigs)
		GO browser
		Annotation
Gene Association	Gene Symbol	EST ID
	Sequence ID	GO annotation
	Unigene Number	Blast hits
Blast	Query (protein/DNA)	Standard blast output [216]
	Database	
Annotations	Keyword	Blastx hits in the searched protein databases
	Sequence ID	Assigned GO terms
Contig Viewer	Contig ID	Schematic overview of contig assembly
		Composing ESTs' IDs
		Contig sequence

Containing a total of 12,709 unique sequences, this EST database probably represents a large part of the desert locust CNS transcriptome. However, the order of magnitude of the full transcriptome is currently unknown, since the desert locust genome has not been sequenced yet. The average length of the EST sequences indicates that there is a relatively high degree of high quality sequence information available per transcript. This information will prove to be useful in bioinformatic analyses and their downstream applications, such as homology searches, prediction of the biological function of the encoded products, design of primers for ‘Rapid Amplification of cDNA Ends’ (RAcE) or for ‘quantitative real-time reverse transcriptase polymerase chain reaction’ (qRT-PCR) analysis and production of microarrays for transcriptome-wide expression profiling studies.

#### Blastx searches in protein databases

The *S. gregaria* sequences were used as a query for blastx searches in the *Anopheles gambiae*, *Tribolium castaneum*, *Drosophila melanogaster*, *Apis mellifera*, *Caenorhabditis elegans*, *Homo sapiens* and the NCBI nr.aa protein databases. These searches produced 4,891 hits in the *A. gambiae* database, 4,780 hits in the *T. castaneum* database, 4,693 hits in the *D. melanogaster* database, 4,735 hits in the *A. mellifera* database, 3,612 hits in the *C. elegans* database, 4,492 hits in the *H. sapiens* database and 5,779 hits in the nr.aa database. An important fraction of these hits consists of (hypothetical) proteins without known function. Furthermore, in the protein databases for various insect species with a completely sequenced genome (*A. gambiae*, *T. castaneum*, *D. melanogaster*, *A. mellifera*), no obvious orthologs were found for *ca.* 60% of the *S. gregaria* EST sequences. This observation may be explained by the fact that EST data do not necessarily cover the entire protein encoding region of a gene. Also, the applied search criteria need to be rather stringent to avoid mistakes in the annotation process. Moreover, locusts are phylogenetically distant from these holometabolous insect models. It can also not be excluded that there may be many species-specific genes that are absent in these model species. These considerations underline the need for additional, functionally analyzed hemimetabolous/orthopteran insect genome/transcriptome data, which may add to the current knowledge of homologous or species-specific genes in desert locusts and other Hemimetabola.

#### Gene Ontology annotations

**Figure 2:**
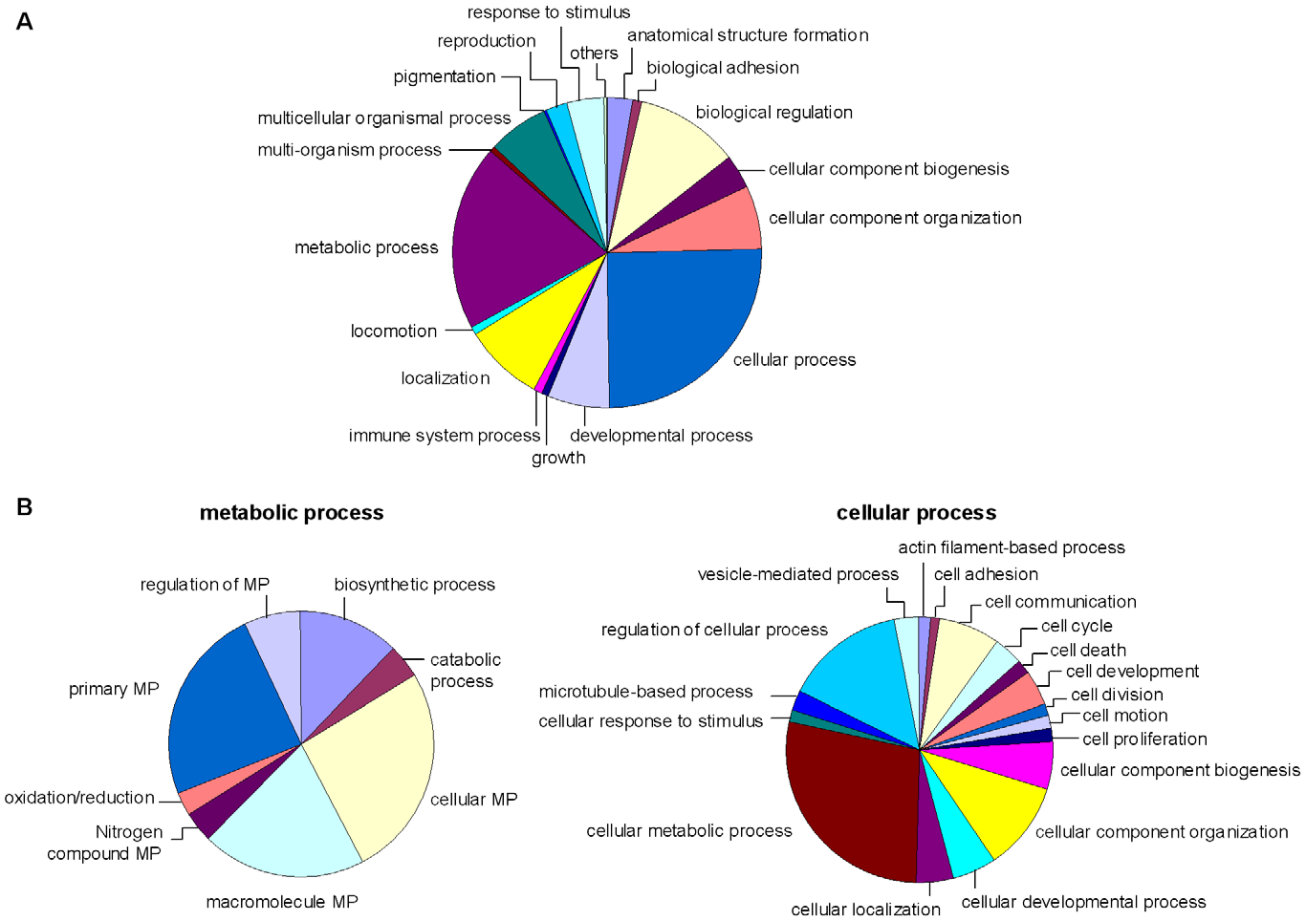
Second level GO distribution of the *S. gregaria* EST sequences (*Biological Process*). Distribution of the *S. gregaria* EST sequences in the major subclasses of the main ontology *Biological Process* (A) and a more detailed overview of sequence distribution in the two subclasses *Metabolic process* and *Cellular process* (B).

In the functional annotation process, 3,887 *S. gregaria* sequences were classified according to the GO vocabulary [33]. Figure 2A shows the distribution of EST sequences among the different subcategories (level 2) of the main ontology *Biological Process*. The best represented GO-groups were *Cellular Process* and *Metabolic Process*. To obtain a more detailed view on the functional differentiation in these GO groups, an overview of the subgroups is presented (Figure 2B), showing that nearly 60% of the ESTs within the GO group *Metabolic process* are classified in *Primary metabolic process* and *Cellular metabolic process*. With regard to cellular processes, more than 50% of these ESTs are classified in three subgroups: *Cellular metabolic process*, *Regulation of cellular process* and *Cellular component organization*.

Next, the distribution of GO-annotated transcripts, classified under the main ontology *Biological Process*, was analyzed in more detail. For all ESTs the GO-term corresponding to the lowest node in the GO hierarchy with an annotation score above the threshold (>45) [34], [35] was used to calculate 100 GO-terms representing the highest number of ESTs. As shown in Figure S1, numerous ESTs were classified in groups describing processes occurring in virtually all cell types, such as cell cycle, transcription/translation events, metabolic processes and transport. Nevertheless, many other ESTs are predicted to be involved in processes such as development and functioning of the nervous system, reproduction, determination of life span and growth.

It is not very surprising that many sequences are classified under metabolic and cellular processes. These are more general GO terms which comprise basic processes, needed to maintain a living organism. Of particular interest are those ESTs that are predicted to be involved in processes such as development and functioning of the nervous system, reproduction, determination of life span and growth. Study of these transcripts may provide information about how the CNS controls reproduction, growth and adult life span and how these processes are regulated in a phase-dependent way.

A classification of EST sequences according to the GO vocabulary [33] represents a convenient starting point for analysis of large amounts of data, such as microarray data. In addition, a GO-based classification also proves to be useful when studying a certain biological process, molecular function or cellular component represented by a specific GO term. For example, *S. gregaria* EST sequences classified under a specific term are easily retrieved from the database and may add to the current knowledge of the studied process. However, the GO terms are only ‘labels’ and should not be taken for granted. Therefore, the user should remain critical when performing GO-based analysis of data. Assignment of GO terms is not only based on experimental evidence and is very often derived from homology searches. In case of the latter, the GO terms have been transferred from homologous sequences to the newly annotated sequence (these assignments are mostly based on a certain score that considers degree of similarity to the homologous sequence, evidence code of how the GO term has been assigned to the homologous sequence, *etc*…). However, the biological function of the homologous sequences may for example not be evolutionary conserved. Also when focusing on a specific tissue or condition, it should be considered that the GO terms assigned to a specific factor may apply to biological roles in other tissues or conditions. It can be summarized that GO terms offer a good starting point for analyzing large sets of sequence data, but that the further analysis process should be performed critically in order to eliminate potential noise disturbing the biological relevance of the data.

#### Comparison to LocustDB

At present, another locust EST database is publicly available. It encompasses 12,161 unique sequences from *L. migratoria* (LocustDB) [25], [26]. Although it is similar in its total transcript sequence number output, this other database was generated from different tissue sources. Whereas LocustDB contains EST data derived from primary (*i.e.* non-normalized) cDNA libraries for head, hind leg, midgut and whole organisms (5^th^ larval stage), the novel *S. gregaria* database entirely focused on transcript information from the CNS (3–5^th^ larval stage and adults). Therefore, it is not surprising that this different approach has been responsible for a very different representation of locust transcript sequences. This is nicely illustrated by the result of comparative blastn searches between both databases showing that only 4,189 sequences (*ca.* 1/3) can be considered as orthologs (cut-off at E-value <1E-10) occurring in both databases. A ‘Blast2GO’ analysis of both databases showed that in total only 5,701 of the 20,681 unique locust (either *L. migratoria* or *S. gregaria*) sequences can be annotated, indicating that the (possible) function of the majority of locust sequences remains unknown. That there is little overlap between two different EST databases may have various reasons. First, as mentioned before, *S. gregaria* and *L. migratoria* belong to different subfamilies within the family of Acrididae. Therefore, the phylogenetic distance between these two species may explain part of the observed lack of homology. Second, the two databases were derived from different tissue sources. By focusing on the CNS, the *S. gregaria* EST database is more specialized, whereas the *L. migratoria* database may be more general. Third, EST data represent only partial sequences of most transcripts. Moreover, the average EST sequence length is larger for the *S. gregaria* sequences. Therefore, it is not unlikely that identification of orthologous sequences may be hampered by a lack of overlapping sequence information. Fourth, EST databases generally do not cover the complete transcriptome of the respective tissues and species. And finally, it should also be emphasized that, in contrast to LocustDB, the *S. gregaria* EST database is derived from a normalized cDNA library, thereby compensating for very abundant transcripts and increasing the odds of sequencing less abundant ones, for which the orthologs may not have been included in the *L. migratoria* database.

To further compare the functional variety of the transcript data represented in both locust EST databases, the distribution of the annotated sequences in the different *Biological Process* GO classes was calculated (where each sequence is classified under the most detailed GO term). The number of sequences linked to a particular class was used to rank the ‘100 best represented’ classes (Figures S1 and S2). Gene products involved in processes occurring in virtually all cell types, such as *Transport*, general metabolic processes (including the GO-classes *Oxidation reduction*, *Proteolysis* and *Metabolic process*) and transcription/translation events (including the GO-classes *Ribosome biogenesis* and *Translation*) are well represented in both databases. On the contrary, the distribution of transcripts in biological processes occurring in specific tissue types tends to be more dependent on the cDNA source of the respective database. The GO-groups *Carbohydrate/starch metabolic process* and *Sucrose metabolic process*, as well as *Digestion* and *Catabolic protein processes* are highly enriched in the *L. migratoria* database (Figure S2). Transcripts associated with the CNS (e.g. *Axon guidance*, *Nervous system development* and *Synaptic transmission*) are predominantly present in the *S. gregaria* EST database (Figure S1). In addition, sequences classified under *Spermatogenesis* and *Growth* are also more represented in the desert locust EST database. This observation can probably be explained by the fact that these processes are either directly or indirectly regulated by factors produced by the CNS and/or by the fact that many factors having a role in development also have a function in the CNS.

It can be summarized that the current generation of *S. gregaria* ESTs adds a very significant amount of novel information, when compared to previously available transcript sequence data from other insect species. It should also be emphasized that, in addition to the phylogenetic distance between *S. gregaria* and *L. migratoria*, there are several technical differences between the two locust databases that may account for the observed lack of homology between the ESTs of both species. Generally, GO-annotated sequences that are linked to the function of a specific tissue/organ are differentially distributed among both databases, providing a complementary set of locust transcriptome information.

### 2. Neuronal signaling and signal transduction components

Virtually all ongoing physiological processes in a living organism are initiated or regulated by a signal from the environment or from other cells. Depending on the incoming signal, a specific receptor (cell-surface or intracellular receptor) is activated and subsequently induces a downstream signal transduction pathway, leading to the cellular response. There are numerous signal transduction pathways, leading to an abundance of cellular responses and cross-talk between different pathways is possible. Signaling pathways make use of a range of molecular components, which may require activation in order to transfer the signal to downstream effectors. The CNS is expected to contain a large number of transcripts coding for components that are involved in neuronal signaling and signal transduction. For a first analysis of the contents of the *S. gregaria* EST database, we therefore have searched for sequences coding for compounds predicted to play a role in these processes. Here, we give an overview of the result of this analysis.

**Figure 3:**
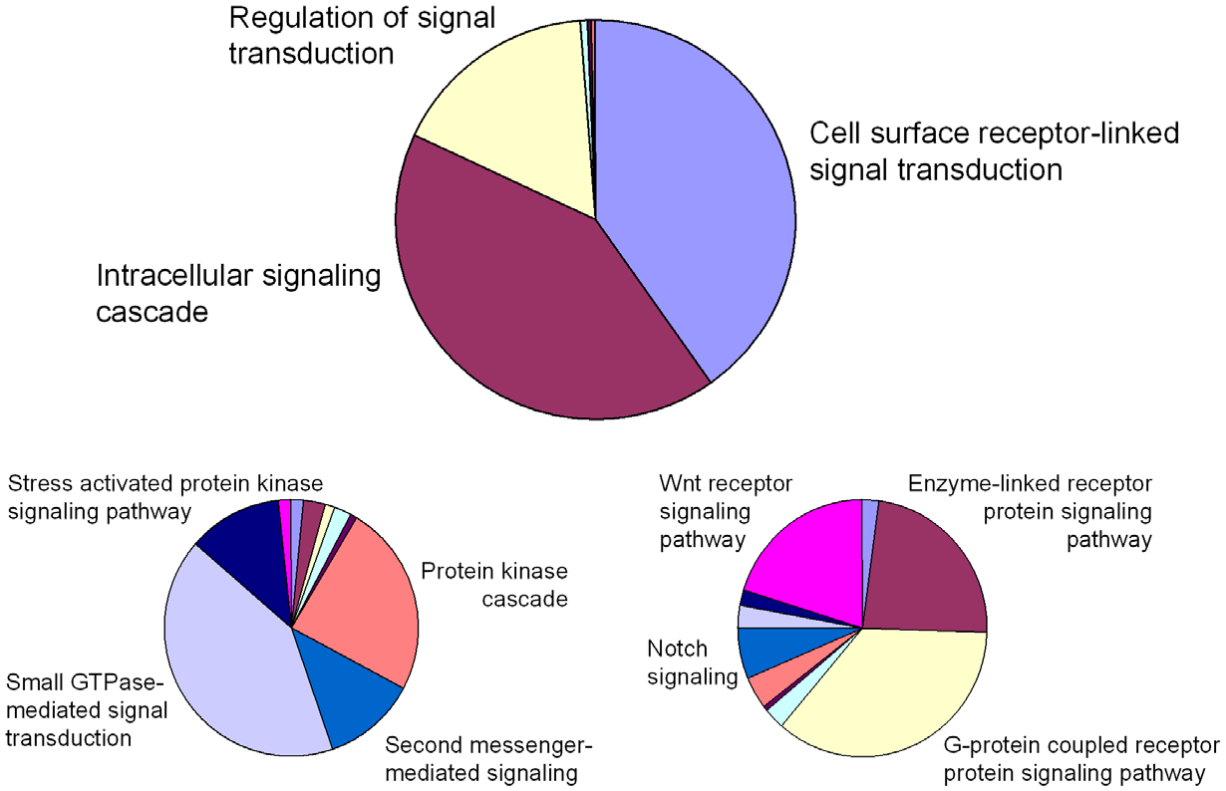
GO distribution of the *S. gregaria* EST sequences classified under *Signal Transduction*. Distribution of the *S. gregaria* EST sequences classified under the GO term *Signal transduction* and a more detailed overview of the sequence distribution in the two subcategories *Intracellular signaling cascade* and *Cell surface receptor-linked signal transduction*.

Of the sequences classified in the database under the GO term *Cellular process*, 108 are associated with *Signal transduction* (*Biological process > Cellular process > Cell communication > Signal transduction*). When all existing GO terms, at all levels, are considered, the biological process of signal transduction is the second best represented GO category in the *S. gregaria* EST database. The majority of sequences classified under the GO term *Signal transduction* can be divided under three GO terms, namely *Cell surface receptor-linked signal transduction*, *Intracellular signaling cascade* and *Regulation of signal transduction* (Figure 3). Since signal transduction in the nervous system entails more aspects than the ones classified under this specific GO term, this overview is extended towards neuronal signaling processes. Therefore, several transcripts that are predicted to be involved in *Peptide hormone processing* or in *Biogenic amine synthesis*, as well as more than twenty neuropeptide precursors, which have been annotated manually, will also be discussed. A list with the sequence IDs of components discussed in the context of (neuronal) signaling is made available in Table 2 and Table S1 (neuropeptides).

**Table 2:** Sequence ID and annotation of EST sequences associated with neuronal signaling.

Cell surface receptor-linked signal transduction	
*G protein-coupled receptor protein signaling pathway*	
LC.1391.C1.Contig1536	opsin
LC01037B2B08.f1	short neuropeptide F receptor
LC01003X1D07.r1	orexin receptor
LC02006X1H05.f1	Methuselah-like
LC01008B2B10.f1	Methuselah-like
LC01013A1E06.f1	putative serotonin receptor
*Neurotransmitter receptor activity*	
LC.1031.C1.Contig1170	nicotinic acetylcholine receptor alpha subunit
LC.907.C1.1039	N-methyl-D-aspartate glutamate receptor
LC01009A2C01.f1	GABA-A receptor
LC01019B2E05.f1	GABA-B receptor
LC01049B1E11.f1	metabotropic glutamate receptor

Neurotransmitters and (neuro)peptides	
*Biogenic amine synthesis*	
LC01054B2B09.f2	dopa decarboxylase
LC.243.C1.Contig314	tyramine beta hydroxylase
LC01006B2E02.f1	tryptophan hydroxylase
*Peptide hormone processing*	
LC.2118.C1.Contig2277	furin 2
LC.381.C1.Contig436	neuroprotein 7B2 precursor
LC.1129.C1.Contig1270	angiotensin converting enzyme
LC02007B2B06.f1	angiotensin converting enzyme
LC.1302.C1.Contig1447	endothelin converting enzyme
LC.3441.C1.Contig3579	endothelin converting enzyme
LC01071B1B03.f1	endothelin converting enzyme
LC.410.C1.Contig495	STE24 homolog

Sequence ID and annotation for the *S. gregaria* EST sequences encoding GPCRs and the EST sequences classified under the GO-terms *Neurotransmitter receptor activity*, *Biogenic amine synthesis* and *Peptide hormone processing*.

#### Cell surface receptor-linked signal transduction (GO:0007166)

More than 80% of the uniques involved in signal transduction through cell surface receptors are predicted to play a role in enzyme-linked receptor protein signaling, Notch and Wnt signaling or G protein-coupled receptor (GPCR) signaling (Figure 3). Given its importance in neuronal signaling (while the former may be mainly involved in developmental processes), the latter category is now discussed in more detail.

A very important group of cell surface receptors are the seven transmembrane segments containing GPCRs [36]–[38]. Many GPCRs play a role in sensory perception and typical ligands are odor molecules, pheromones or light-sensitive components [39], [40]. Other GPCRs function as receptors for a wide range of hormones and neurotransmitters [41], [42], indicating that these receptors have versatile functions in a large variety of physiological processes. The fact that human GPCRs are the target for a vast number of drugs is yet another indication for their importance [43].

In the *S. gregaria* database several ESTs were annotated to be involved in *GPCR signaling pathways* (GO: 0007186). An opsin-like GPCR was found in the EST database that displays similarity to the previously identified desert locust opsin-1 protein, which is involved in perception of long wavelength light [44].

In addition to short neuropeptide F (sNPF) receptors from *D. melanogaster* [45], [46], the red fire ant *Solenopsis invicta* [47] and the mosquito *A. gambiae* [48], [49], a novel putative sNPF receptor has now been identified from the *S. gregaria* database. Recently, two sNPF peptides were identified from both *S. gregaria* and *L. migratoria*, and these peptides were shown to be widespread in the locust neuroendocrine system [50]. In *D. melanogaster* sNPF regulates food intake and body size [51] and it was shown to affect growth by a functional interaction with extracellular regulated mitogen-acitvated kinase (ERK)-mediated insulin signaling [52]. Furthermore, in the fruit fly it also has an effect on foraging, social and aggressive behavior [53], [54].

Another gene product from the *S. gregaria* database shows significant homology with predicted orexin receptors from *Pediculus humanus corporis* (Kirkness *et al.*, unpublished), *Nasonia vitripennis*, *T. castaneum* and *A. mellifera*. Orexin is a mammalian neuropeptide involved in the control of several processes, including food intake. Interestingly, a *Bombyx mori* homolog of mammalian orexin receptors was recently demonstrated to be capable of responding to *Manduca sexta* allatotropin (AT), for which the receptor had long remained unknown [55]. AT has recently been associated with food intake control in insects [56]. Since solitarious and gregarious locusts show different foraging strategies, it is possible that AT and its receptor may be regulators of this phase-dependently regulated process. Furthermore, AT was initially described, at least in some insect species, as a potent stimulator of juvenile hormone (JH) synthesis [57]. JH has previously been demonstrated to induce certain phase-specific characteristics [58], again suggesting that AT and its receptor may possibly be involved in the control or establishment of phase-dependently regulated physiological processes. This hypothesis deserves further attention.

The *S. gregaria* EST database also contains Methuselah (Mth)-like GPCRs. Mth was first identified from *D. melanogaster* as a secretin-like receptor [59], belonging to the B-family of GPCRs [38]. Interestingly, downregulation of Mth in the fruit fly resulted in an increased life span [60]. Although Stunted was originally identified as the ligand for Mth and mutants also resulted in increased life span [61], it was recently demonstrated that Mth displays a promiscuous response to several non-homologous peptides, including the *Drosophila* sex peptide and the newly identified ‘Serendipitous Peptide Activator of Mth’ (SPAM). However, mutations in Mth did not result in changes in sex peptide-induced behavior, questioning the biological significance of its Mth activation [62].

Finally, based on sequence similarity to (putative) serotonin receptors from *Culex quinquefasciatus*, *Aedes aegypti* and *T. castaneum*, one of the *S. gregaria* ESTs has been annotated as a putative serotonin receptor, while it also has some similarity to adenosine receptors. As serotonin has been demonstrated to be a crucial mediator for gregarization behavior [13], study of this putative receptor and its activated signaling pathway(s) might advance our knowledge of locust phase transition and could constitute a useful basis for the development of a new generation of products for a ‘locust-specific’ pest control. Although some other neurotransmitter receptors are also GPCRs, they are discussed in the following paragraph.

Neurotransmitter receptors are, unlike the previous GO terms, classified as a separate GO term (GO:0030594: *Neurotransmitter receptor activity*) under the main GO ontology *Molecular Function*. In general, two types of neurotransmitter receptors can be discriminated, namely ionotropic and metabotropic receptors. Whereas ionotropic receptors form an ion channel pore upon activation, metabotropic receptor activity results in indirect activation of a plasma membrane ion channel through a series of intracellular events [63], [64]. Transcripts encoding both types of neurotransmitter receptors are found in the desert locust EST database. These include an NMDA (N-methyl-D-aspartate) type of ionotropic glutamate receptor, which is co-activated by glutamate and glycine [65]. Second, the database also contains a metabotropic glutamate receptor 1, a GPCR which stimulates NMDA receptor activity through activation of phospholipase C, a rise in intracellular Ca^2+^ and activation of protein kinase C [66]. In addition, sequences resembling an ionotropic ‘γ-aminobutyric acid’ (GABA)-A and a metabotropic GABA-B receptor have been annotated. Finally, a transcript is present that corresponds to the previously reported α-subunit of the nicotinic acetylcholine ionotropic receptor [67], [68].

Although receptor transcripts tend to be rare and are generally poorly represented in EST databases, this overview demonstrates that the *S. gregaria* EST database nevertheless contains several receptor encoding sequences, which may be involved in a range of important processes in the CNS. Study of the proteins encoded by these transcripts will provide us with new functional information which may constitute a scientific basis for the development of novel pest control agents or strategies. Insect pest control is nowadays often based on the use of chemicals that interact with a very limited number of neuronal targets, such as acetylcholinesterase, the voltage-gated sodium channel, the acetylcholine receptor, or the GABA receptor [69]. However, due to resistance phenomena and an increasing demand for ecologically considered pest control strategies, there is a constant need for new and more species-specific compounds (generating fewer side effects). Because GPCRs play such an important role in novel drug discovery programmes of pharmaceutical companies, they may constitute interesting targets in the context of pest control strategies as well. Therefore, the study of GPCRs may possibly broaden the range of species-specific pest control targets. The wider the range of targets, the more easily resistance phenomena can be avoided. However, not all GPCRs will prove to be excellent targets for pest control. An important step forward in locust pest management would be to identify specific receptor ligands that are capable of disturbing the process of phase change.

#### Biogenic amine synthesis (GO:0042401)

Neuronal signaling processes involve neurotransmitter (and/or neuromodulator) molecules responsible for communication between neurons or communication between neurons and other target cells. Neurotransmitters are synthesized in neurons and vary in structure, ranging from single amino acids and mono-amines to peptides (which may also function as hormones). In what follows, an overview is given of transcripts related to the synthesis of biogenic amines and the processing of neuropeptide precursors. In addition, sequences corresponding to neuropeptide precursors are also presented.

Various neurotransmitters are biogenic amines, which are derived from amino acids. Several enzymes involved in the synthesis of these biogenic amines were predicted from desert locust EST sequences. Both dopamine and octopamine are synthesized from L-tyrosine. The dopamine synthesis pathway starts with hydroxylation of L-tyrosine to L-dihydroxyphenylalanine (L-DOPA) by the enzyme L-tyrosine hydroxylase [70], [71]. L-DOPA is subsequently converted to dopamine by removal of the carboxyl group by DOPA decarboxylase [71], [72]. Synthesis of octopamine is initiated by decarboxylation of L-tyrosine (L-tyrosine decarboxylase) [73]. Next, the resulting L-tyramine (which may also function as a neuronal signal [74]) is hydrolyzed by tyramine β-hydroxylase and the end product is termed octopamine (the role of octopamine in locusts and other arthropods was recently reviewed by Verlinden *et al.* [75]). The precursor for serotonin, L-tryptophan, is converted by subsequent actions of tryptophan hydroxylase and 5-hydroxytryptophan decarboxylase into 5-hydroxy-L-tryptophan and serotonin, respectively [76]–[78]. Sequences coding for tyramine β-hydroxylase, DOPA decarboxylase and tryptophan hydroxylase orthologs are present in the *S. gregaria* database.

#### Peptide hormone processing (GO:0016486)

Neuropeptides generally require processing from a larger precursor polypeptide to become biologically active. The factors that aid in this processing process are proprotein or prohormone convertases and many of these are members of the subtilisin family. Subtilisin-like convertases generally cleave the peptide precursor at dibasic (Lys-Arg or Arg-Arg) or monobasic (Arg) amino acid sites [79]. Furin, which is a subtilisin-type of enzyme, cleaves specifically at Arg-Xaa-Yaa-Arg sites (Xaa: any amino acid, Yaa: Arg or Lys) [80], [81]. Substrates for furin include polypeptide hormones, growth factors, growth factor receptors, neuropeptides and viral envelope glycoproteins. Furin has been characterized in mammalian species, but also in several invertebrate species. The desert locust EST database includes a homolog for *D. melanogaster* furin 2.

In addition, the desert locust EST database includes a transcript encoding a factor which is homologous to the mammalian neuro-endocrine protein 7B2. Although this factor does not possess protein processing activity itself, it is a regulator of the maturation of prohormone convertase 2 (PC2) [82]–[85]. PC2 transcript levels in mammals are most abundant in neuro-endocrine cells [86]–[89], which explains why it is involved in the processing of important peptide hormone precursors. *D. melanogaster* homologs for PC2 (dPC2) and 7B2 (d7B2) have also been identified, and it was demonstrated that dPC2 requires d7B2 for both maturation and secretion [90]. Interestingly, the desert locust transcript for the 7B2 homolog appears to be upregulated in isolated-reared animals, as demonstrated in the present study (*cf. infra*).

Insect homologs for the zinc metalloproteases ‘angiotensin converting enzyme’ (ACE) and ‘endothelin converting enzyme’ (ECE) [91], [92] have also been suggested to play a role in peptide processing and/or degradation. Mammalian ACE is needed to process angiotensin I into angiotensin II, which is a peptide displaying vasoconstrictor activity and hence increases blood pressure. This conversion involves release of the C-terminal dipeptide from angiotensin I, which explains why ACE is also termed dipeptidyl carboxypeptidase [93]. A homolog of mammalian ACE was previously characterized in *L. migratoria*, and an *S. gregaria* EST encodes an almost identical enzyme. Immunolocalization studies in *L. migratoria* already demonstrated the presence of ACE in neurosecretory cells from brain and suboesophageal ganglion, in the storage part of the *corpora cardiaca*, in both the *nervi corporis cardiaci* and the *nervi corporis allati* and in the testes [94], [95]. In three groups of neurosecretory cells, ACE was found to co-localize with locustamyotropins, suggesting that these factors may be ACE substrates. Locustamyotropins extended with Gly-Arg-Arg or Gly-Lys-Arg can indeed be hydrolyzed by recombinant *D. melanogaster* ACE [94]. Furthermore, *L. migratoria* ACE was also demonstrated to be involved in degradation of locustatachykinin-1 [96]. Finally, qRT-PCR studies revealed that *L. migratoria* ACE transcripts are found in virtually all tissues. Interestingly, ACE transcript levels in haemocytes increase in response to bacterial lipopolysaccharide administration, suggesting that ACE might play a role in immunity as well [97]. An insect ECE was first identified from *L. migratoria*. In vertebrates, ECE catalyzes the conversion from big endothelin into the vaso-active endothelin-1 by cleaving the -Trp_21_-Val- bond [98]. In *L. migratoria*, ECE activity was demonstrated in neuronal membranes [99] and in the reproductive system, although specific substrates for ECE remain to be identified. Three ESTs were predicted to encode an ECE-like protein, one of which encodes a protein almost identical to *L. migratoria* ECE.

Another putative processing enzyme represented in the desert locust EST database is a STE24 endopeptidase. This type of enzyme was first characterized in the yeast *Saccharomyces cerevisae*. It was shown to remove aaX from the C-terminal CaaX-motif found in the a-factor, which is a mating pheromone (C: cysteine, a: aliphatic amino acid, X: any amino acid) [100], [101]. Generally, CaaX-containing proteins are processed as follows: 1) a prenyl group is attached to the cysteine residue, 2) the aaX group is proteolytically removed and 3) the cysteine α-carboxyl group is methyl esterified. The prenylated and methyl esterified cysteine residue is now the new C-terminus and may form a hydrophobic membrane anchor. At present, STE24 endopeptidases have also been identified in bacteria, archaea, and virtually all eukaryotic kingdoms. Although STE24 endopeptidases have been predicted from insect genome data, no studies of their substrates have been performed so far.

#### Neuropeptide precursors

Neuropeptides are a versatile class of signaling molecules produced in the nervous system. Mostly, the bioactive peptide is processed from a larger precursor polypeptide (*cf.* reviews on neuropeptides and their precursors in locusts and other insects [4], [5], [102], [103]). An *in silico* analysis of the novel *S. gregaria* EST database resulted in the (manual) annotation of more than twenty neuropeptide precursor encoding transcript sequences (Table S1). Thirteen sequences appear to cover the entire reading frame (from start to stop codon) coding for the precursor polypeptide, while nine others represent partial sequences coding for a large, but still incomplete portion of the peptide precursor. Six of these precursor transcript sequences (present as four complete and two partial sequences in the EST database) were previously identified in the desert locust, *S. gregaria*, by conventional cDNA cloning strategies, *i.e.* the ones encoding the following peptides: adipokinetic hormone I (AKH-I) [104], short and long ion transport peptides (ITP-S and ITP-L) [105], neuroparsins 1 and 2 (NP-1 and NP-2) [106], and allatostatins (AST-A) [107]. The current study thus reveals sixteen novel sequences (eight complete and eight partial ones) for peptide precursor transcripts in the desert locust *S. gregaria*. Moreover, the characterization of these precursor sequences also results in the prediction of several (putative) neuropeptides which had as yet not been identified before (neither at the protein nor at the nucleic acid level) in *S. gregaria*. An overview of these novel desert locust peptides is given in Table 3.

**Table 3:** Overview of the newly predicted *S. gregaria* neuropeptides.

Neuropeptide	ID	Amino acid sequence *S. gregaria*
AST CC	LC.407.C1.Contig492	SYWKQCAFNAVSCFamide
Burs-β	LC01070B1E02.f1	VVRAPLEVDGIDKLDIEFRCCRCQWACNSQVQPSVTTPTGFLKECYCCRESFLRERTVTLSHCYDPDGARLTAEGTATMDIRLREPAECKCFKCGDFSR
CCHa	LC01024B1A06.f1	GCMAFGHSCFGGHamide
DH	LC.1479.C1.Contig1625	MGMGPSXXIVNPMDVLRQRLLLEIARRRLRDAEEQIKANKDFLQQIamide
GPA	LC.3116.C1.Contig3268	MVPPSSRSALHFFALAVALCLSAVSAGMDGERDAWEKPGCHRVGHTRKISIPDCIEFPITTNACRGFCESWSVPSALNTLRVNPHQAITSIGQCCNIMETEDVEVRVMCLDGPRDLVFKSAKSCQCYHC
HrTH	LC.3853.C1.Contig3980	QVTFSRDWSPamide
ITG	LC.817.C1.Contig945	ITGKVASFNHI
NPF	LC.1768.C1.Contig1921	QQAAADGNKLEGLADALKYLQELDRY**YSQVARPRFamide** *
TK-2	LC.108.C1.Contig162	APLSGFYGVRamide
TK-3	LC.108.C1.Contig162	APQAGFYGVRamide
TK-4	LC.108.C1.Contig162	APSLGFHGVRamide
TK-5	LC.108.C1.Contig162	APLLGFHGVRamide
TK-6	LC.108.C1.Contig162	APLRGFQGVRamide
TK-7	LC.108.C1.Contig162	ALKGFFGTRamide
TK-8	LC.108.C1.Contig162	GNT***KK***APVGFYGTRamide **
Predicted neuropeptides that do not show homology to any known peptide		
PVK-4	LC.2414.C1.Contig2580	KGLVANARVamide
PVK-5	LC.2414.C1.Contig2580	DSLWFGPRVamide
MT-2	LC.2414.C1.Contig2580	TSSLFPHPRIamide
MT-3	LC.2414.C1.Contig2580	SLRL***R***LPAAAWLAAGDVGNGKGDFTPRLamide **
PVKDP	LC.2414.C1.Contig2580	AGLGQDETRAGTK
NLP-1	LC.1768.C1.Contig1921	YLASLVRSHGLPYPLT
NLP-2	LC.1768.C1.Contig1921	EDDGPGEI
NLP-3	LC.1768.C1.Contig1921	NVGALARNWMLPSamide
NLP-4	LC.1768.C1.Contig1921	ASDDDQEVD
NLP-5	LC.1768.C1.Contig1921	YLASVLRQamide
NLP-6	LC.1768.C1.Contig1921	HLGSLAKSGMAIH
NLP-7	LC.1768.C1.Contig1921	FLGVPPAAADYamide
NLP-8	LC.1768.C1.Contig1921	HIGALARLGWLPSFRAASA***R***SG***R***SAGSRSamide **

This table shows an overview of the newly predicted *S. gregaria* neuropeptides (*i.e.* which had not been identified previously, neither at the protein nor at the nucleic acid level) and the sequence ID of the precursor encoding transcript. It needs to be emphasized that some of these peptides are predicted on the basis of possible cleavage sites within the precursor but do not show homology to any known peptide. Their presence and function(s) *in vivo* have not been demonstrated yet. In addition, the precursor sequences are derived from EST sequence information and a limited degree of sequencing errors cannot be fully excluded. Abbreviations: AST CC: allatostatin double C; Burs-β: bursicon β-subunit; DH: diuretic hormone; GPA: glycoprotein hormone α; HrTH: hypertrehalosemic hormone; NPF: neuropeptide F; TK: tachykinin (TK 2–4 had previously been demonstrated by means of mass spectrometry, but their complete amino acid sequence had so far not been completely determined [5]); PVK: periviscerokinin; PPDP: PVK precursor-derived peptide; NLP: neuropeptide-like peptide. The nomenclature of CCHa is based on the fact that this neuropeptide has two conserved cysteine residues and an amidated histidine residue. The nomenclature of Apis ITG is based on a pattern of three amino acid residues in the sequence.

*: The amino acid residues printed in bold represent the truncated form of NPF, which had previously been demonstrated by means of mass-spectrometry [50].

**: A possible cleavage site within the predicted peptide is printed in bold italics. Different cleavage forms of a certain peptide may occur in a tissue-dependent manner. For instance, TK-8 may occur as GNTKKAPVGFYGTRa or APVGFYGTRa; MT-3 may occur as SLRLRLPAAAWLAAGDVGNGKGDFTPRLa or LPAAAWLAAGDVGNGKGDFTPRLa or GDFTPRLa. For NPLP-8, different cleavage patterns might perhaps result in HIGALARLGWLPSFRAASARSGRSAGSRSa, LGWLPSFRAASARSGRSAGSRSa, AASARSGRSAGSRSa, HIGALARLGWLPSFRAASA, SGRSAGSRSa, HIGALARLGWLPSFRAASARSa or SAGSRSa.

The complete precursor sequences for the adipokinetic hormones, AKH I and AKH II, were retrieved from the *S. gregaria* EST database. The AKHs were among the first insect peptide hormones to be described, and the first fully characterized AKH was AKH-I from *L. migratoria* [1]. AKHs are important in releasing energy from reserves by increasing lipid and trehalose levels in the haemolymph [108]–[110]. Hypertrehalosemic hormone, HrTH, is a related neuropeptide which stimulates trehalose synthesis and release from the fat body. The migratory locust HrTH peptide has previously been identified by Siegert [111]. A transcript sequence encoding the entire HrTH precursor occurs in the *S. gregaria* EST database and reveals that both locust HrTH peptides are identical.

The allatostatins (AST-A family) were originally isolated from brain tissue of the cockroach *Diploptera punctata* as peptides inhibiting JH biosynthesis by the *corpora allata* [112], [113]. Besides their allatostatic activity, these peptides appear to be pleiotropic in function [114], [115]. Members of the AST-A peptide family have also been characterized in the desert locust [116] and their precursor cDNA, which codes for ten distinct AST-A related peptides (also designated as ‘schistostatins’) has been cloned [107]. After the discovery of additional neuropeptides with JH biosynthesis inhibiting activities in a variety of insect orders, ‘allatostatins’ were further classified in three distinct families (AST-A, B and C) based on sequence characteristics. Recently, ‘AST-CC’ neuropeptides were identified as a novel group of peptides displaying some sequence similarities to AST-C-type allatostatins. In the current study, a transcript sequence coding for a complete AST-CC precursor is found in the desert locust EST database. It is highly similar to a partial *L. migratoria* sequence that has been detected in LocustDB [117], [118].

Allatotropin (AT) was initially purified from the moth *M. sexta* as a 13-residue amidated peptide that strongly activates JH synthesis [57]. A locust member of the AT peptide family was identified by Paemen *et al.* [119] as a myotropin from *L. migratoria* male accessory gland extracts (the initial name of this peptide was *Lom*-AG-MT I). Recently, this AT was also found in several tissues of *S. gregaria* and sequenced partially by means of mass spectrometry [5]. By analyzing the transcript sequence in the EST database, the full AT precursor has now been unveiled in the desert locust.

Insectatachykinins (TKs), also designated as tachykinin-related peptides, form an evolutionary conserved family of brain-gut peptides characterized by a common C-terminal sequence -FXGXRamide (X = variable amino acid residue) [102], [103]. The first members of this family were isolated as myotropic peptides from the CNS of *L. migratoria* [120], [121]. The desert locust database contains a transcript sequence encoding a precursor that contains a secretory signal peptide and nine distinct TK related peptides. The peptides are flanked by dibasic cleavage sites (mostly Lys-Arg or KR) and each peptide has a C-terminal amidation signal (Gly or G). Five of these peptides have previously been demonstrated in desert locust nervous tissue extracts by means of mass spectrometry and were termed *Scg*-TK-1-4 (two peptides on the precursor are identical and correspond to *Scg*-TK-4) [5]. The amino acid sequences of *Scg*-TK-2-4 could in that study only partially be determined, but the current study reveals that these peptides are identical to the corresponding TKs from *L. migratoria*. A partial *L. migratoria* TK precursor sequence was previously retrieved from Locust DB [122].

Neuropeptide Y (NPY), the most abundant neuropeptide in the mammalian nervous system, is a highly conserved 36 amino acid neuromodulator [123]. NPFs are considered as invertebrate homologs of vertebrate NPY. Whereas the vertebrate NPYs have a C-terminal amidated tyrosine (Y) residue, NPFs end with an amidated phenylalanine (F). NPFs have been found in several invertebrate phyla, such as flatworms [124], [125], mollusks [126], [127] and insects [128]. The *L. migratoria* EST database, LocustDB [25], [26], provided the first evidence for the existence of NPF in locusts. In addition, a complete NPF precursor encoding transcript has now also been identified from the desert locust EST database. The obtained precursor sequence encodes (long) NPF, which is nearly identical to *L. migratoria* NPF. A truncated form of NPF (YSQVARPRFamide) has previously been demonstrated in both locust species [50].

Ion transport peptide (ITP) was first characterized from the desert locust, *S. gregaria*, as a peptide that stimulated transport of Cl^−^ ions across the hindgut epithelium [129], [130]. Two different locust cDNAs were cloned, encoding a short (ITP-S) and a long variant of ITP (ITP-L) [105], [131]. Both transcript sequences (coding for the complete precursors) were also encountered in the desert locust EST database. ITP-S and ITP-L peptides are highly similar in their N-terminal region, but differ in their C-terminal part. In addition, ITP-L has four more amino acid residues. Both locust ITP precursors also contain an ITP co-peptide between the signal peptide and ITP. Although this co-peptide has been confirmed by mass spectrometry, its function is as yet unknown [5], [132].

Neuroparsin (NP-1) was initially identified from *L. migratoria* as a peptide having antigonadotropic, as well as several other pleiotropic functions [133]–[139]. Later, a similar peptide was also characterized in *S. gregaria* [140]. Cloning studies in the desert locust further revealed the existence of four distinct NP precursors (NPPs), two of which (NPP-1 and NPP-2) were more prominently expressed in the CNS [106], [141]. At present, NPs and NP-like peptides have meanwhile been identified from several other invertebrate species [142]. Interestingly, NPs display sequence similarity to the hormone binding domain of vertebrate ‘insulin-like growth factor binding proteins’ (IGFBPs) and a recombinant NP was demonstrated to be capable of interacting with the *S. gregaria* insulin-related peptide *in vitro* [143]. Moreover, desert locust NPP transcripts have been shown to be expressed in a (isolated/crowded-reared) phase and reproduction cycle dependent manner [144], [145]. The *S. gregaria* EST database contains a complete and a partial sequence coding for the neuroparsin precursors NPP1 and NPP2, respectively.

Another transcript in the *S. gregaria* database was found to encode a complete CCHamide-like neuropeptide precursor. CCHamides were first identified in the silkworm *B. mori* [146] and their name refers to two conserved cysteine residues (CC) and a C-terminal amidated histidine residue (Ha).

The last neuropeptide for which a complete precursor sequence was retrieved shows sequence similarity to a peptide previously identified from *A. mellifera* by mass spectrometry [147]. It was termed *Apis* ITG peptide because of its first three amino acids, but so far nothing is known about its physiological function.

In this study, a large part of the ‘crustacean cardioactive peptide’ (CCAP) precursor transcript sequence was identified from the *S. gregaria* EST database. The CCAP was first identified from the crab *Carcinus maenas* as a heart contraction accelerating peptide [148]. CCAP has also been isolated from the locusts *L. migratoria* and *S. gregaria* [116], [149]. In locusts, CCAP has been shown to act as a pleiotropic factor, stimulating hindgut and oviduct contractions [149], [150] and triggering the release of AKH from *corpora cardiaca* [116].

Another cardioactive peptide identified from *M. sexta* was designated as ‘Cap-2b’ [151]–[155]. This peptide was later shown to be a member of the periviscerokinin (PVK) family (so-called because of their presence in abdominal perivisceral organs) [156], [157]. PVKs have also been identified from locust abdominal ganglia and perivisceral organs and display similarity to the peptides derived from the *D. melanogaster capability* gene encoded precursor [158]. EST sequence information reveals that the precursor encoded by an *S. gregaria* transcript sequence contains the periviscerokinins *Scg*-PVK-2 (GLLAFPRVa) and *Scg*-PVK-3 (DGAETPGAAASLWFGPRVa). The periviscerokinin/myotropin-like peptide *Scg*-MT-2 (TSSLFPHPRLa) was suggested to be encoded by the same precursor [158]. However, we only found the nearly identical sequence TSSLFPHPRIa in the current precursor. Nevertheless, it should be emphasized that *Scg*-MT-2 was initially identified by means of mass spectrometry which does not make the distinction between leucine and isoleucine residues. In addition, the PVK precursor encodes four (extra) newly predicted peptides. Two of these show a leucine at position n−7, an arginine at position n−2 and a C-terminal amidation which are typical characteristics of insect PVKs [159]. These two peptides are therefore termed *Scg*-PVK-4 and *Scg*-PVK-5. Another peptide contains the -FXPRLamide C-terminus that is typical for mytropins and is designated as *Scg*-MT-3. The last peptide shows no homology to any known insect peptide and is designated as ‘PVK precursor-derived peptide’ (PVKDP). In addition, a blast search revealed that this *S. gregaria* transcript displays highest sequence similarity to the (computationally predicted) capability-like precursor from the aphid, *A. pisum*.

Locust diuretic hormone (DH) was isolated from brain and *corpora cardiaca* as a peptide that stimulated urine production in *L. migratoria* [160], [161]. An *S. gregaria* transcript sequence encoding a precursor for a closely related DH is found in the EST database. Both locust peptides seem to be identical, although two amino acids of the *S. gregaria* DH could so far not be determined. Insect DH displays a motif that is typical for members of the vertebrate corticotropin releasing factor (CRF) family [162]. Because of the dissimilar gene structures, it is however still under discussion whether insect DHs and vertebrate CRFs are true orthologs. Similarly to vertebrate IGFs, the bioavailability and bioactivity of CRFs is regulated by binding proteins. CRF binding proteins (CRF-BPs) and related factors appear to be remarkably well conserved in metazoan evolution and occur in both the deuterostomian and protostomian lineages. Because of the similar gene structure and conserved cysteine pattern, the CRF-BP-like proteins of insects have been described as true orthologs of vertebrate CRF-BPs [163]. Although the biological role of insect DHs and vertebrate CRFs seems to differ, the above example may represent an extra argument suggesting that DH and CRF are both part of an evolutionary conserved system. In this context, it is interesting to observe that a CRF-BP ortholog is also predicted from the *S. gregaria* EST database (LC.2107.C1.Contig2266).

The ‘ovary maturating parsin’ (OMP) is a locust gonadotropic peptide that is produced in the *pars intercerebralis*, hence the name ‘parsin’ [140],[164]. The identification of its precursor cDNA has been unsuccessful until this study. Two *S. gregaria* EST sequences encode two (slightly) different OMP precursors, hereby representing the first (partial) OMP precursor transcript sequences identified in insects. Each of the two identified precursors encodes a different isoform of OMP, the difference between both mainly resides in an insertion of three amino acids (Pro-Ala-Ala or PAA) [140].

Bursicon is a neurohormone initiating cuticular tanning and wing spreading immediately after eclosion of an adult fly [165]–[168]. The active hormone is a heterodimer consisting of two cysteine knot containing subunits [169], [170]. Although bursicon subunits have been identified in several insect orders and even in other invertebrate phyla [171], this study is the first to reveal a partial peptide sequence of the β-subunit of bursicon in locusts.

Members of the glycoprotein (GP) hormone family, such as vertebrate thyroid-stimulating hormone (TSH) and gonadotropins [follicle-stimulating hormone (FSH), luteinizing hormone (LH) and chorionic gonadotropin (CG)], are also heterodimeric factors that consist of two cysteine knot containing subunits, an α (GPA) and a β subunit (GPB) [172]–[176]. Later, based on vertebrate genome data, an extra α (GPA2) and β subunit (GPB5) have been predicted [177], [178]. Interestingly, orthologs of GPA2 and GPB5 have also been identified in invertebrates [177]. In *D. melanogaster*, a heterodimer of these subunits was shown to be capable of activating the ‘leucine-rich repeat-containing GPCR 1’ (dLGR1) [179]. In the *S. gregaria* EST database, a transcript sequence is found that encodes a GPA-like peptide, which displays highest similarity to putative GPAs from *P. humanis corporis* and *T. castaneum*.

Furthermore, a partial *S. gregaria* transcript sequence appears to encode a ‘neuropeptide-like precursor 1’ (NPLP1)-like sequence. Previously, three neuropeptides showing no homology to other known neuropeptides were identified from a *D. melanogaster* larval CNS extract. It appeared that these peptides were all encoded by the same precursor, which was consequently termed ‘neuropeptide-like precursor’ (NPLP) [180]. Later, similar precursors were identified from *A. mellifera* [181], *T. castaneum* [182], *A. gambiae* [183] and *Neobellieria bullata* [184]. Although the predicted *S. gregaria* precursor displays putative hallmarks of an NPLP precursor, comparative sequence analyses of NPLP precursors have revealed substantial heterogeneity among different species, impeding unambiguous sequence-based identification of NPLP orthologs. The eight predicted *S. gregaria* NPLP-encoded peptides are referred to as ‘neuropeptide-like (precursor derived) peptides’ (NLP) 1–8.

The last decades, a wide range of neuropeptides have been isolated from locust nervous tissue extracts, illustrating the advantage of the big size of locusts for neurobiological and endocrinological studies [1]–[5]. However, the lack of gene sequence information often proved to be a drawback for the application of novel techniques. The above findings show that this EST database constitutes a very important step to bridge this gap allowing for the implementation of new post-genomic research strategies (e.g. peptidomics, proteomics and transcriptomics) techniques. This is also nicely illustrated by a recent neuropeptide search performed in the shrimp *Litopenaeus vannamei*. Based on the availability of an EST database, peptide precursors were predicted and by combining the obtained information with mass spectrometry, the *in vivo* occurrence of many *L. vannamei* peptides could be confirmed [185].

### 3. Genes differentially regulated in the two phases

**Figure 4:**
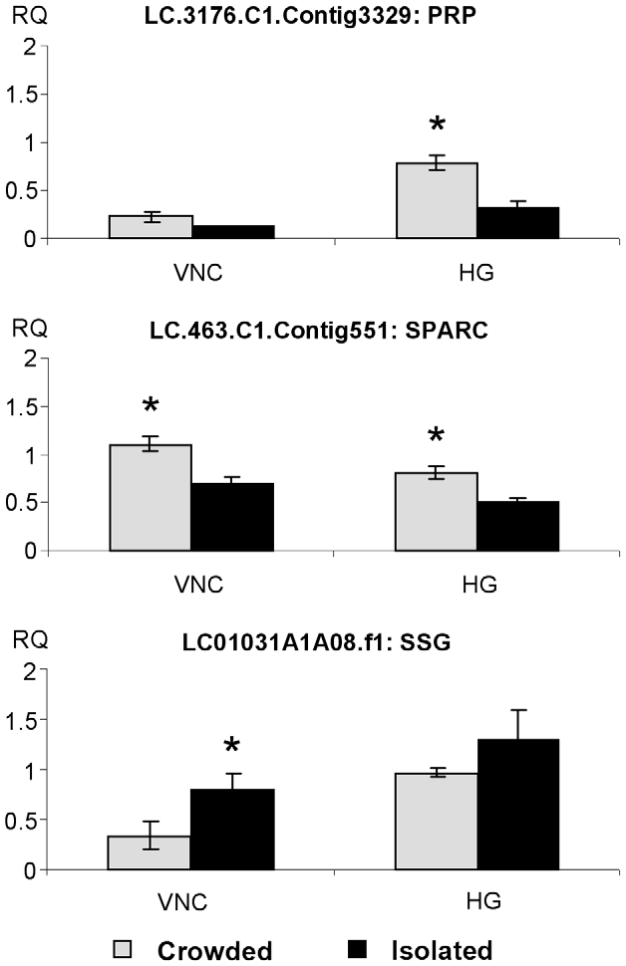
Relative transcript levels for PRP, SPARC and SSG in desert locusts in the two phases. Relative transcript quantity (RQ) for PRP, SPARC and SSG in isolated- and crowded-reared desert locust ventral nerve cords (VNC) and head ganglia (HG). Results were obtained by analyzing four independent pooled samples of ten individuals per condition and are represented as means ± standard error. Statistical analysis consisted of a Student's t-test for comparing two independent groups. Significantly higher transcript levels (p<0.05) are indicated by an asterisk (*).

**Figure 5:**
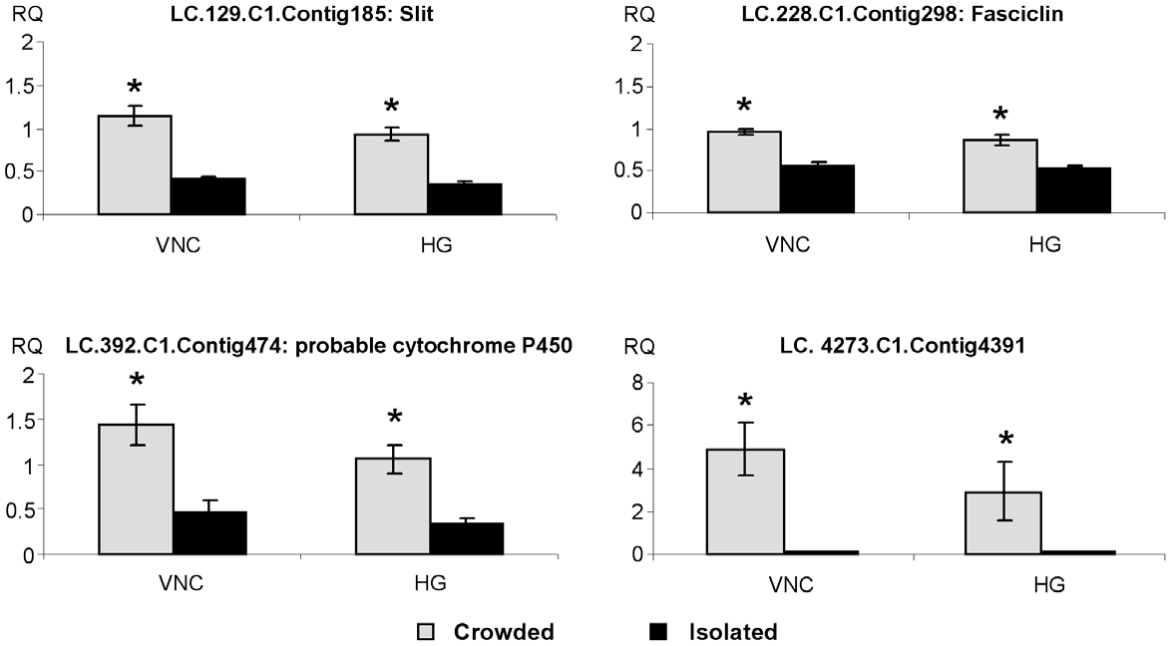
Relative transcript levels for genes showing higher expression levels in nervous tissue of crowded-reared locusts. Relative transcript quantity (RQ) for genes showing higher expression levels in crowded-reared desert locusts (VNC: ventral nerve cord, HG: head ganglia). Results were obtained by analyzing four independent pooled samples of ten individuals per condition and are represented as means ± standard error. Statistical analysis consisted of a Student's t-test for comparing two independent groups. Significantly higher transcript levels (p<0.05) are indicated by an asterisk (*).

**Figure 6:**
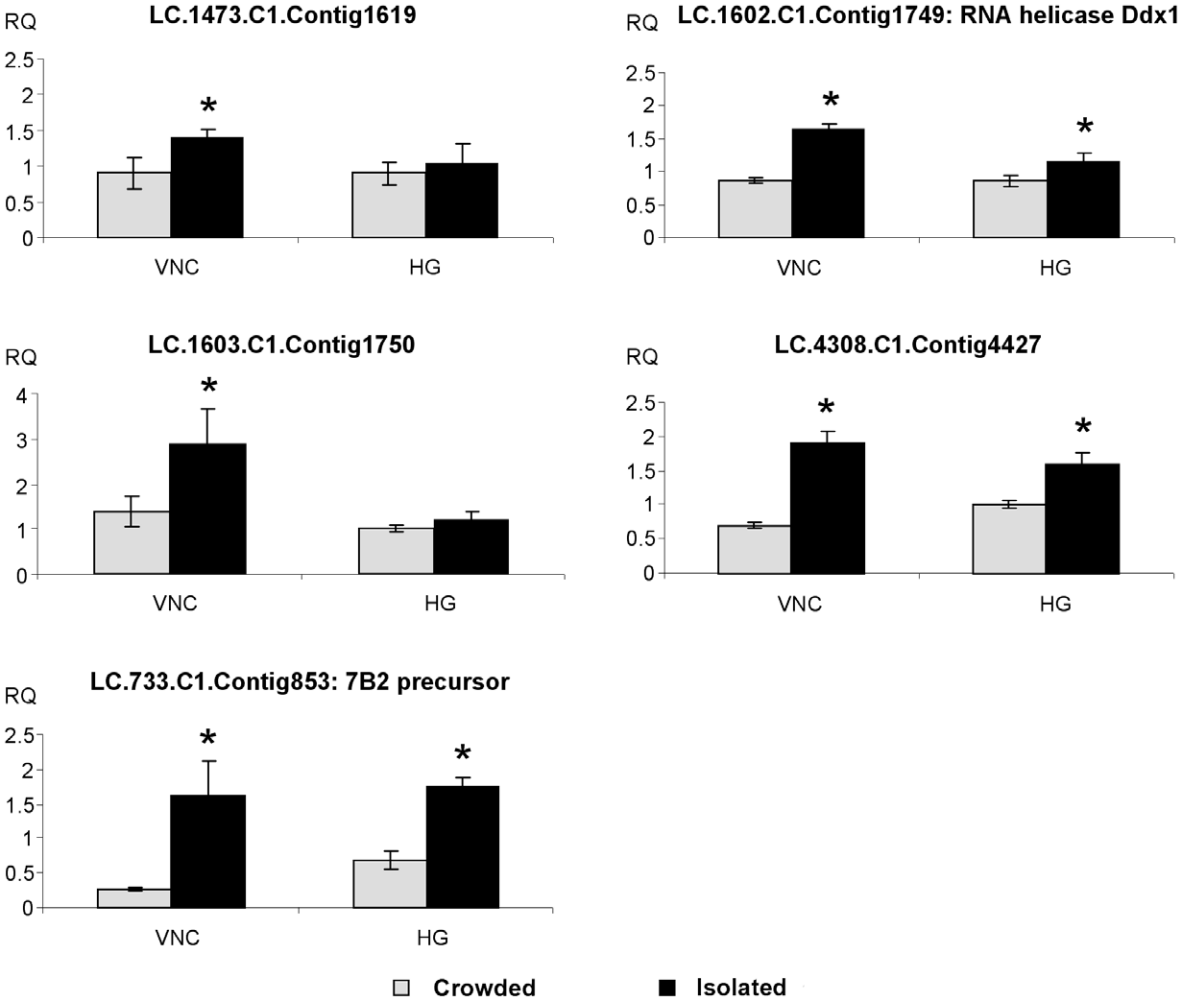
Relative transcript levels for genes showing higher expression levels in nervous tissue of isolated-reared locusts. Relative transcript quantity (RQ) for genes showing higher expression levels in isolated-reared desert locusts (VNC: ventral nerve cord, HG: head ganglia). Results were obtained by analyzing four independent pooled samples of ten individuals per condition and are represented as means ± standard error. Statistical analysis consisted of a Student's t-test for comparing two independent groups. Significantly higher transcript levels (p<0.05) are indicated by an asterisk (*).

The novel EST database will constitute a valuable tool for future studies analyzing and comparing transcript profiles in desert locusts under different developmental or physiological conditions. In our lab, microarray studies are being prepared for a transcriptome-wide analysis of phase-dependent gene expression in the desert locust. Meanwhile, since specific sequence tags had been incorporated in the cDNA inserts derived from isolated- and crowded-reared animals during library construction, an analysis of contigs consisting of at least five tagged ESTs resulted in the selection of an initial set of transcripts for a qRT-PCR analysis of their relative abundance in isolated- and crowded-reared animals’ nervous systems (Table 4). Three additional transcripts, for which corresponding EST sequences were identified in the *S. gregaria* database, were also evaluated as (putative) positive controls in this study (Table 5). Relative levels for all transcripts which were statistically confirmed by qRT-PCR as differentially expressed in isolated- and crowded-reared locusts are shown in Figures 4, 5 and 6.

**Table 4:** Overview of the contigs that have been selected by tag-based evaluation for qRT-PCR analysis.

EST ID	C	I	Annotation	Forward primer (5′→3′)	Reverse primer (5′→3′)
***Crowded>Isolated***					
**LC.228.C1.Contig298**	**9**	**0**	**Fasciclin(-like) precursor**	GAATCACTTGGTGGGCCTCTT	CCATATGAATGCGACCCTCAT
LC.308.C2.Contig390	8	0	*No annotation*	CAAAAATCTGTGCCAAGGAACTG	GCGCTTCAACAACAGCAATC
LC.393.C1.Contig477	8	0	Similar to CG12163 (Cys-protease inhibitor)	CAAGCGTGAGATTACGGAAATACA	AGGTAGTGCTGCTCGTCTCGTT
LC.1955.C1.Contig2112	7	0	Similar to T-complex protein 1 subunit gamma	TGCGTGTGGTGCTACTATTGTG	TGTTCCCACGTCATCTTCCTT
**LC.4273.C1.Contig4391**	**7**	**0**	***No annotation***	GCATAGGAGAGTGAAGCATTCACA	ACAAGAATGCAGACAAAAACTACACA
LC.446.C2.Contig534	5	0	Similar to 14-3-3 protein (leonardo protein)	CGTGTCAGTTGGCGAAACAG	CTTCGTTTAGCGTATCCAGTTCAG
**LC.129.C1.Contig185**	**6**	**1**	**Slit homologue**	GGCGCACCTCAAATTGGA	TCCACCGTGAAGCTGTCTTG
LC.1849.C1.Contig2006	6	1	Signal peptidase complex subunit 2	AAACATTGTGTTTGCATGGGTAAG	GGCTGCGCTTCCTTGCT
**LC.392.C1.Contig474**	**5**	**1**	**Probable cytochrome P450**	GAGGTGAACCGTGGAGAAAGTT	CGGCTCGCTGTGAAGGA
***Isolated>Crowded***					
**LC.1602.C1.Contig1749**	**0**	**15**	**RNA helicase Ddx1**	TGTCCTAGTCGAGGACGGAATT	TGCAACAGCCTCCTTCATCA
LC.587.C1.Contig691	0	11	G-protein gamma subunit	CGTACGGCTGCATTAAATTCTG	AGGAACGGGCGACTGAATC
**LC.1473.C1.Contig1619**	**0**	**7**	***No annotation***	GCTGTGACATTTCTGGCCTCTT	TTCATTAGAGGGATACTCTTTCAAGCTA
LC.312.C1.Contig394	0	7	Glutamine synthetase	CGCGCGAATCTGCAAGA	TGTCGGGCTGCGGAAGT
**LC.4308.C1.Contig4427**	**0**	**7**	***No annotation***	AGTCATTCTGAGAGAGACAAAGTTTCTTAT	AACACTGCAATTCGCTTCGA
**LC.1603.C1.Contig1750**	**0**	**5**	***No annotation***	CCCCCTGGTGGACAGTCAT	TGCCAATACGTGCACAGAATC
**LC.733.C2.Contig853**	**1**	**5**	**Similar to 7B2 precursor**	ACCTCATTCAGCGCCAAAAT	CCCAGCCATGCTGGAGTCT

The different columns show the sequence ID of the contig, the number of tags referring to the origin (C: crowded-reared; I: isolated-reared) of the composing ESTs per contig, the annotation of the sequence and the primers that have been used for the qRT-PCR assays. The contigs printed in bold black are those for which the corresponding transcript levels were significantly different in nervous tissue of crowded- and isolated-reared locusts. The transcript levels corresponding to the contigs printed in normal black were not found to be significantly different in nervous tissue from locusts in the two phases.

**Table 5:** Overview of the EST/contig sequences corresponding to PRP, SPARC and SSG.

EST ID	Annotation	Reference	Forward primer (5′→3′)	Reverse primer (5′→3′)
LC.3176.C1.Contig3329	Phase-related peptide	[186], [188]	CCGTCTGAAATTCAAAGATGGAA	CGCGACGCATATGGTATCC
LC.463.C1.Contig551	SPARC (secreted protein, acidic, rich in cystein)	[187]	GGCGAGTCCGCACTACAACT	CACCATCCTCTGCTCCTTGAA
LC01031A1A08.f1	Solitary phase specific gene	[187]	CGACTGACGCCTGATTTTCC	CACAGTGACTGGGCGTGAGA

Overview of the EST/contig sequences corresponding to PRP, SPARC and SSG and the primers that have been used for their qRT-PCR assays.

#### Confirmation by qRT-PCR of previous studies

‘Phase-related peptide’ (PRP) and ‘secreted protein acidic and rich in cysteine’ (SPARC) transcript (which was initially selected by differential display PCR) more abundantly occur in crowded-reared desert locust haemolymph and CNS, respectively [186]–[188], while the ‘solitarious phase specific gene’ (SSG) transcript was previously identified by differential display PCR and found to be more abundant in isolated-reared locust CNS [187]. In the present study, qRT-PCR indeed confirmed that the transcripts encoding SSG and SPARC are more abundant in the nervous system of isolated- and crowded-reared locusts, respectively (Figure 4). While PRP has been detected as a highly abundant peptide in crowded-reared locust haemolymph extracts, only relatively low quantities were found in extracts of isolated-reared animals [186], [188]. In line with these initial observations, which were all performed at the peptide/protein level, this study now shows that the corresponding transcript levels are also significantly higher in nervous tissue from crowded-reared desert locusts. Although their biological role(s) remain(s) to be elucidated, PRP and SSG transcripts may serve as molecular markers for phase transition.

SPARC is an extracellular matrix-associated Ca^2+^-binding glycoprotein and has pleiotropic functions in vertebrates, mainly in embryonic tissues and adult tissues undergoing remodeling [189]–[191]. These functions include regulation of cell shape [192], [193], cell adhesion [194], [195], cell cycle [196], extracellular matrix [197], [198], cell proliferation [196], [199] and cell migration [200], [201]. Furthermore, SPARC was demonstrated to interact with certain growth factors, thereby influencing the cellular response to these factors [194], [199], [202]. Interestingly, a SPARC-like factor was shown to have a very particular effect on neuronal cell migration, regulating radial glia-guided neuron migration in the rat cerebral cortex [203]. Although SPARC has been identified in many insect species, its involvement in neuronal migration has hitherto not been studied.

#### Differentially expressed genes: crowded > isolated

By means of qRT-PCR, the levels of nine other transcripts were shown to significantly differ between crowded- (Figure 5) and isolated-reared (Figure 6) animals, either in head ganglia (HG) or ventral nerve cord (VNC) or in both CNS parts. That not all tested transcripts show significant differences in relative abundance in the isolated- and crowded-reared locust CNS may have two possible reasons. First, it needs to be emphasized that the approach of tag-based evaluation of contigs should rather not be seen as a truly quantitative method. It is also only of use for the most abundant transcripts (for which contigs could be created). In this context it needs to be emphasized that the *S. gregaria* EST database has been derived from normalized cDNA libraries, thereby compensating for very abundant transcripts. Since only a limited number of tagged ESTs is available per contig the factor coincidence should not be completely excluded. Therefore, based on this initial analysis, a selection of 16 transcripts was made, for which the expression level in both phases was further investigated by qRT-PCR. In the end, the qRT-PCR results allowed us to formulate more solid statements about differences in expression levels of the tested genes between locusts reared in the isolated or crowded condition. Second, the qRT-PCR assays were performed starting from nervous tissue derived from 4- to 10-day old adults. Since sequence information in the EST-database has been derived from locusts in several developmental stages, a stage-dependency of certain phase-related differences should not be excluded. Nevertheless, we feel that this independently obtained evidence strengthens our conclusions.

In the context of the previous paragraph, it may also appear of interest to notice that two transcripts which show significantly higher levels in crowded-reared desert locusts, code for factors predicted to be involved in development and/or modeling of the nervous system. One of these transcripts encodes a locust homolog of Slit, a factor known to be involved in axon guidance in both vertebrates and invertebrates. To assure that growing neurons connect the two brain halves in *D. melanogaster*, Slit will prevent these neurons from crossing back over the midline [204]. Dimitrova *et al.* [205] also demonstrated that fruit flies mutant in Slit or in its receptor Robo (roundabout), displayed space-filling neurons with longer, but less branched dendrites. Recently, midline signaling molecules, including Slit and Robo, were shown to play a crucial role in the formation of a neural map by dendritic targeting of motor neurons in the *D. melanogaster* nervous system [206]. The second transcript encodes a homolog of fasciclin-I, which was shown to play a role in neuron path finding. This cell adhesion molecule is involved in certain cell-cell interactions necessary for development of the nervous system. In addition, it was shown previously that fasciclin plays an important role in axonogenesis during embryonic brain development in *S. gregaria* [207]. These findings suggest that the gregarious CNS is possibly in a more plastic state than the solitarious CNS. It will thus be of interest to evaluate whether SPARC also has a role in neural plasticity in *S. gregaria*.

Crowding-induced aggregation behavior of locusts implicates learning (habituation) and memory related processes [208]. Both non-associative learning and memory require the acquisition and retention of neuronal representations of new informational input and are typically associated with neuroplasticity. While a small brain size and a relatively simple neuronal organization in combination with a short life span used to be common arguments for rejecting an important role for brain plasticity in insects, several neurogenetic studies have overthrown this assumption (as reviewed by Dukas. 2008 [209]). Accordingly, we have characterized at least three transcripts (*slit*, *fasciclin-1* and *SPARC*) that may be involved in the modeling and/or organization of the CNS and are upregulated in adult crowded-reared locusts, as compared to their isolated-reared conspecifics. It should also be noted that the *S. gregaria* EST database contains 210 transcripts classified under the GO term *Nervous system development* and that 179 (85%) of these transcripts are involved in neurogenesis and CNS development. Therefore, the necessary information is now available to perform a wide-scale comparative transcriptomic analysis of the molecular mechanisms underpinning the neurobiological dynamics of the locust CNS during phase transition.

Although the outcome of this analysis is much awaited, accumulating evidence points to a crucial role for serotonin. Not only was this biogenic amine demonstrated to be a necessary and sufficient trigger for gregarization behavior [13], it had earlier been shown to be a regulator of neuroplasticity in a number of different animals. In the *M. sexta* olfactory system, serotonin has an effect on excitability of neurons and it was suggested to play a role in structural plasticity of these neurons (more specifically in neuron growth and establishment of new neuron connections) [210]. The latter is also supported by previous observations, describing a positive effect of serotonin on the growth of olfactory neurons *in vitro* [211]. In the marine mollusk *Aplysia* it was shown that the learning process of gill and siphon withdrawal reflexes is accompanied by formation of new connections between siphon sensory neurons and their target cells. Intriguingly, this process of new synapse formation could also be mimicked by serotonin, which was moreover demonstrated to influence the number of fasciclin II-like cell adhesion proteins [212]. Although functional serotonin-fasciclin interactions have hitherto not been studied in insects, it is plausible that both factors may also act together in regulation of neuroplasticity in insects.

We also identified a putative cytochrome P450 encoding transcript as being more abundantly present in the nervous tissue of crowded-reared locusts. The superfamily of cytochrome P450 proteins comprises heme-containing enzymes that oxidize diverse organic substrates [213]. However, no specific functional information is as yet available about this transcript or the protein it encodes. Therefore, future studies will be required to determine its role in phase-dependently regulated processes.

#### Differentially expressed genes: crowded < isolated

A transcript that is less abundant in crowded- than in isolated-reared locusts, encodes a DEAD-box protein Ddx1 homolog. DEAD-box proteins are involved in RNA splicing and early translation events [214], [215]. This may possibly indicate that some phase-related differences arise from the very basic level of RNA processing and translation. Accordingly, a similar study in *L. migratoria* predicted that transcripts classified under the GO-terms *Ribosome* (*Cellular Component*), *Nucleic acid metabolism* and *Protein biosynthesis* are more abundantly present in isolated-reared locusts [25].

Another transcript that was significantly more common in isolated-reared locusts encodes a homolog of the PC2-supporting protein 7B2 (*cf. supra*) [82]–[85]. Neuropeptides and peptide hormones are typically derived from larger precursors and act as regulators in a wide range of physiological processes, such as growth, development, reproduction, ecdysis, diuresis, feeding behavior and metabolism. Since the activity of prohormone processing enzymes is essential for the production of (most) bioactive peptides, the neuropeptidergic regulation of (many) physiological processes may also be indirectly linked to the level of precursor processing. Differences in 7B2 transcript levels in the two locust phases may therefore be one of the underlying mechanisms leading to a differential regulation of a wide range of physiological processes during phase transition.

For several other transcripts that are significantly more abundant in isolated- or crowded-reared locusts, it is not yet clear what their role in phase transition might be or why they are more abundant in one of the two phases. Many sequences in the database could not (yet) be annotated, since clearly orthologous sequences could not (yet) be identified. For example, LC.4273.C1.Contig4391, strongly upregulated in crowded-reared locusts, and LC.4308.C1.Contig4427, increased in isolated-reared locusts, are certainly worth further investigation, but at present there is no information about their possible biological activities. Nevertheless, several of the newly identified phase-dependently regulated transcripts may constitute promising (candidate) markers for monitoring the process of locust phase transition.

It needs to be mentioned that the solitarious (isolated) locust colony was reared in isolation for many generations. By separating the isolated- and crowded-reared locusts for such a long time we aimed at establishing genuine long-term phases (when only studying early behavioral changes a lower number of isolated generations can be applied). Since the colonies for both phases were quite large (hundreds of individuals per generation for the isolated-reared colony), genetic drift and inbreeding phenomena were expected to remain limited. In addition, by washing egg pods from crowded-reared females and introducing the newly emerged hoppers into the isolated-reared colony, ‘fresh blood’ has from time to time been brought in. Nevertheless, genetic drift and inbreeding cannot be fully ruled out and, at this time, the role of genetic divergence *versus* environmental effects cannot be conclusively determined [216]. We intend to study these phenomena in more detail via microarray and/or deep sequencing analyses. Nonetheless, we are convinced that further exploration and thorough analysis of the EST database will most certainly contribute to future investigations on the process of phase polyphenism and its accompanying neuroplasticity, as well as to many other research themes in the biology of the desert locust.

In summary, by developing an *S. gregaria* EST database we met the need for extra hemimetabolous insect transcriptomic data. The current report describes the construction of an *S. gregaria* EST database containing 12,709 unique transcript sequences. In addition, we demonstrated that construction of this database did not result in a high degree of redundancy of locust transcriptomic data. For now, analysis of the database already allowed us to functionally annotate 3,887 sequences, many of which are annotated as involved in neuronal signaling and signal transduction. Finally, several genes displaying significantly differential transcript levels in isolated- and crowded-reared desert locusts were identified. Interestingly, some of these are predicted to be involved in development and modeling of the nervous system. These observations contribute to the view that density-dependent behavioral plasticity in locusts is not only defined by innate signaling pathways, but represents a more sophisticated adaptation for coping with complex differences in environmental situations, including neural plasticity.

By specifically focusing on the CNS, this *S. gregaria* EST database will most certainly contribute to future studies unraveling the complex regulation of phase transition and allow to study neuro-endocrine control mechanisms of certain physiological processes. Furthermore, parallel studies focusing on phase polyphenism and factors involved in nervous system development will most probably lead to novel insights in phenomena of neuroplasticity in general.

## Materials and Methods

### 1. Preparation of the central nervous system samples

#### Animals

The *S. gregaria* colony in our laboratory originated from the Aquazoo in Düsseldorf (Germany, 1985). To start their breeding programme, the Aquazoo had collected animals from the field in Nigeria, Africa. Crowded-reared *S. gregaria* were kept under a 13 h light, 11 h dark photoperiod at a temperature which was maintained at 32°C. Each cage also contained at least 1 light bulb (25 Watt) to create a light and temperature gradient within the cage. To sustain the gregarious characteristics the animals were kept in Plexiglas cages (40×32×48 cm) at a density of 500–1000 newly emerged hoppers per cage. They were fed daily with fresh cabbage and oat flakes *ad libitum*.

Isolated-reared locusts were kept individually for 25 successive generations in plastic rearing containers (14×8.5×7.5 cm). They received the same food as the crowded-reared animals. The breeding rooms for isolated-reared locusts have 20 exchanges of air volume per hour, a constant temperature of 32°C and an identical light-dark cycle as in crowd-reared rooms. (For more detailed information on solitarious (isolated) locust breeding, see Hoste *et al.*, 2002 [217].)

The main objective of this project was to determine an as large as possible part of the CNS transcriptome covering different stages. Third to fifth larval instars were selected randomly for dissection to get an equal distribution of age. Adults were staged at eclosion and dissected at regular time intervals (1, 5, 7, 9, 13, 15, 17, 19 and 21 days after eclosion) to cover the sexual maturation and initial reproduction period. In total, head ganglia (HG) and ventral nerve cords (VNC) were dissected from 1,431 animals, distributed as evenly as possible over different developmental stages, sexes and phases.

#### RNA extractions

Desert locust nervous tissues were micro-dissected under a binocular microscope, cleaned and rinsed in Ringer solution (1 L: 8.766 g NaCl; 0.188 g CaCl_2_; 0.746 g KCl; 0.407 g MgCl_2_; 0.336 g NaHCO_3_; 30.807 g sucrose; 1.892 g trehalose; pH 7.2) and immediately collected in RNA*later* solution (Ambion) to prevent degradation. HG and VNC were collected separately. Until further processing, pooled tissue samples (each sample consisted of a certain stage, phase and origin) were stored at −20°C. Samples were added to MagNa Lyser Green Beads (2 ml screw tubes filled with 1.4 mm ceramic beads) and homogenized in the MagNA Lyser instrument (Roche) (6,500 rpm during 30 s). Subsequently, total RNA was extracted from the resulting homogenates utilizing the RNeasy Lipid Tissue Mini Kit (Qiagen), following the manufacturer's instructions. In combination with this extraction procedure, a DNase treatment (RNase-free DNase set, Qiagen) was performed to eliminate potential genomic DNA contamination. Each extraction was followed by a spectrophotometric quantification and quality control with the Agilent 2100 Bioanalyzer (Agilent Technologies). Hence, only those samples were used for which RNA degradation proved to be minimal, thereby excluding contamination with small degraded pieces of RNA during the further workflow. For each stage, sufficient RNA of optimal quality was prepared. All samples were divided into four categories: 1) isolated-reared HG, 2) isolated-reared VNC (thoracic and abdominal ganglia), 3) crowded-reared HG and 4) crowded-reared VNC (thoracic and abdominal ganglia).

### 2. Construction of the EST database

Construction and normalization of cDNA libraries, sequencing and subsequent development of the EST database were performed at the W.M. Keck Center for Comparative and Functional Genomics (University of Illinois, Urbana-Champaign). Normalization of the cDNA library was performed to compensate for very abundant transcripts, thereby increasing the odds of sequencing rare transcripts. Two normalized libraries were constructed using different methods, hence both protocols are described below. The aim was to generate an EST database containing at least 10,000 unique transcript sequences. When the sequencing redundancy for ESTs derived from the first library became higher than 50% before reaching this number, we decided to construct a second normalized library [redundancy was calculated as: 100 * (total number of sequences−number of unique sequences)/total number of sequences]. Both methods involved denaturation of double stranded (ds) cDNA or vector, followed by a hybridization step which was limited in time. The resulting, newly formed ds DNAs were then eliminated, while the sequences that remained single stranded (ss) were further used in the normalization protocol. The common concept of both alternative methods is that abundantly present transcripts will more easily find a complementary sequence, whereas rare transcripts have a much higher probability of remaining ss.

#### Construction of the first normalized library

Poly(A)^+^mRNA was isolated from total RNA using the Oligotex Direct mRNA kit (Qiagen). The poly(A)^+^mRNA was converted to double-stranded (ds) cDNA by using tagged primers (tag underlined) which contain a NotI restriction site. The primers were used as follows (V = A,C,G):

CNS head (HG) isolated-reared:
5′- AACTGGAAGAATTCGCGGCCGCACGCATTTTTTTTTTTTTTTTTTV -3′
CNS body (VNC) isolated-reared:
5′- AACTGGAAGAATTCGCGGCCGCACCGATTTTTTTTTTTTTTTTTTV -3′
CNS body (VNC) crowded-reared:
5′- AACTGGAAGAATTCGCGGCCGCTCGCATTTTTTTTTTTTTTTTTTV -3′
CNS head (HG) crowded-reared:5′- AACTGGAAGAATTCGCGGCCGCTCCGATTTTTTTTTTTTTTTTTTV -3′

Ds cDNAs were size selected (>600 bp). An equal quantity of the cDNA from each of the different samples was pooled, ligated to *EcoRI* adaptors (5′-AATTCCGTTGCTGTCG-3′, Promega #C1291) and digested with *NotI*. This pooled cDNA was then directionally cloned into *EcoRI*-*NotI* digested pBluescript II SK+ phagemid vector (Stratagene). The total number of white colony forming units (cfu) before amplification was 3×10^6^. Blue colonies (empty vectors) were less than 2%.

Normalization of the primary library was performed as previously described by Bonaldo and co-workers [218]. Purified plasmid DNA from the primary library was converted to single-stranded (ss) plasmids and used as a template for a polymerase chain reaction (PCR) by using the T7 and T3 priming sites flanking the cloned cDNA insert. The purified PCR products, representing the entire cloned cDNA population, were used as a driver for normalization. Hybridization between the ss library (50 ng) and the PCR products (500 ng) was carried out for 44 hr at 30°C. By means of hydroxyapatite chromatography [218] unhybridized ssDNA plasmids were separated from hybridized DNA rendered partially ds and electroporated into DH10B cells (Invitrogen) to generate the normalized library. The total number of clones with insert was 2×10^6^. Background of empty clones was less than 2%.

#### Construction of a second normalized library

Poly(A)^+^mRNA from each of the tissues was reverse transcribed into ds cDNA by using the Creator Smart cDNA Library Construction kit (Clontech) following the manufacturer's instructions. The following 5′ tagged oligos (tag underlined) were used for cDNA synthesis:

CNS Head (HG) isolated-reared: Creator SMART IV original
5′ AAG CAG TGG TAT CAA CGC AGA GTG GCC ATT ACG GCC GGG 3′
CNS Body (VNC) isolated-reared: Creator AA
5′ AAG CAG TGG TAT CAA CGC AGA GTG GCC ATT ACG GCC AAG GG 3′
CNS Head (HG) crowded-reared: Creator TT
5′ AAG CAG TGG TAT CAA CGC AGA GTG GCC ATT ACG GCC TTG GG 3′
CNS Body (VNC) crowded-reared: Creator GA
5′ AAG CAG TGG TAT CAA CGC AGA GTG GCC ATT ACG GCC GAG GG 3′

Equal amounts of ds cDNA from each of the tissues were mixed and a total of 300 ng of cDNA was denatured at 98°C for 2 min and allowed to renature at 68°C for 5 hr in 50 mM Hepes (pH 7.5) and 0.5 mM NaCl. Ds cDNAs (i.e. abundant transcripts) were degraded by the addition of Duplex-Specific Nuclease (Evrogen). The ss fraction (which constituted the normalized library) was converted to ds cDNA by specific-suppression PCR as described in the Trimmer-direct kit (Evrogen). The normalized cDNA was directionally cloned into a pDNR-LIB vector (Clontech) and transformed using DH10B electrocompetent cells (Invitrogen). The total number of white colony forming units (cfu) was 2×10^6^.

#### Plasmid isolation, sequencing and sequence processing

Libraries were plated on agar and random clones were picked with the Genetix Q-Pix robot and racked as glycerol stocks in 384-well plates. After overnight growth, bacteria were inoculated into 96-well deep cultures with Luria Bertani Medium and 100 mg/ml of Carbenicillin (libraries cloned in pBluescriptII SK+) or 30 mg/ml of Chloramphenicol (libraries cloned in pDNR-LIB). Plasmid DNA was purified from the bacterial cultures after 24 hr of growth at 37°C with Qiagen 8000 and Qiagen 9600 robots.

Sequencing reactions of the 5′ ends of the inserts (standard T7 primer for libraries cloned in pBluescriptII SK+, primer 5′-CGAGCGCAGCGAGTCAGT-3′ for libraries cloned in pDNR-LIB) were performed using BigDye terminator (Applied Biosystems) on two ABI 3730XL capillary system sequencers (Applied Biosystems). Phred quality scores were calculated for each base call [219], [220]. Bases with a score ≥20 (equivalent to 99% confidence) were considered of high quality. High quality sequence regions were determined by Qualtrim (W.M. Keck Center for Comparative and Functional Genomics, University of Illinois, Urbana-Champaign). In order to maximize the sequence length, some bases with a Phred score <20 were tolerated. Sequences were considered successful if the high quality region was ≥200 bases, otherwise they were termed ‘low quality’. Vector sequences were detected and masked by using Cross_Match (http://www.phrap.org/phredphrapconsed.html). Both vector and low quality sequences were trimmed off the original sequences. Sequences with a length of ≥200 bases after trimming were considered ‘clean’ sequences, otherwise they were called ‘short insert’. Repeat and low complexity sequences were identified and masked by using RepeatMasker Open-3.0 (http://www.repeatmasker.org/). Finally a screen was performed for unwanted sequences such as *Escherichia coli* genome, vector, mitochondrial and viral DNA and ribosomal RNA by using blastn [221]. Sequences having significant similarities with these DNA sequences were considered as contaminants and excluded from the final ‘clean’ sequence set.

#### Contig assembly

If two or more sequences represented the same transcript they were assembled into a ‘contig’. This means that the overlapping EST sequences were aligned and joined in an as complete as possible representation of the corresponding transcript sequence (which is here termed ‘contig’). Assembly of sequences into contigs was performed by using the Phrap software package (http://www.phrap.org/phredphrapconsed.html). When transcripts were represented by only one sequence, the EST was referred to as a ‘singleton’.

### 3. Annotations

#### Blastx searches in protein databases

All obtained EST sequences (contigs and singletons) were used as query for a blastx search [221] in protein databases from *A. gambiae*, *T. castaneum*, *D. melanogaster*, *A. mellifera*, *B. mori* (all of which are insects with a completely sequenced genome), *C. elegans* and *H. sapiens*, and in the ‘National Center for Biotechnology Information’ (NCBI) nr.aa protein database. Sequences producing an e-value<1E-7 were considered a hit.

#### Gene Ontology annotations

All EST information is integrated in an ‘Expressed Sequence Tag Information Management and Annotation’ (ESTIMA) database (http://titan.biotec.uiuc.edu/locust/) [222]. Functional GO annotations [33] of *S. gregaria* sequences in ESTIMA were based on the blastx hits from *A. gambiae*, *T. castaneum*, *D. melanogaster*, *A. mellifera*, *B. mori*, *C. elegans* and *H. sapiens*, which are reference organisms that had previously been GO annotated. These GO annotations can be easily retrieved from ESTIMA.

Apart from ESTIMA, functional annotation of the *S. gregaria* EST sequences was also performed by means of the software package ‘Blast2GO’ [34], [35]. This facilitates high-throughput functional annotation by assigning GO terms [33] to the input sequences. All further GO-based analyses described in this study were performed by means of the ‘Blast2GO’ software. The rationale for doing so was (i) to easily analyze the GO annotations (as ‘Blast2GO’ includes several functionalities for graphical representations and statistical analysis) and (ii) to compare data from *S. gregaria* CNS with publicly available *L. migratoria* EST sequences by performing the same analysis for both, based on the same criteria (*cf. infra*). Functional annotation in ‘Blast2GO’ was performed in several steps. First, a blastx search [221] in the NCBI nr.aa database was performed, and all sequences producing an E-value<1E-3 and a minimal alignment length of 33 residues were considered a hit. Based on the GO annotations for the hit sequences, an annotation score for the candidate GO terms was calculated [34], [35]. This score takes into account the degree of similarity to the blast hits, the evidence code for how the GO term had been assigned to the blast hit sequences, the number of blast hits displaying the GO term and a factor that determines the weight of parent GO terms. If this score was >45, the GO term was assigned to the query sequence. If both a parent and a child term had a score >45, the lowest node in the hierarchy (i.e. the child term, most detailed node) was chosen. Next, all sequences were used as a query to search in the InterPro database [223], [224]. GO terms were derived from the obtained hits and for each sequence they were merged with the already assigned terms. Finally, an ‘Annex’ step was performed, which resulted in an annotation augmentation for the already annotated sequences [34], [35].

#### Study of signal transduction components

A more detailed study of components involved in signal transduction was performed by analyzing ESTs classified under the biological process term *Signal transduction*. The GO term *Signal transduction* is a level 4 term under *Cellular process* (1: *Biological process* >2: *Cellular process* >3: *Cell communication* >4: *Signal transduction*). Given the importance of neuronal signaling in the CNS, a similar analysis was done for sequences classified under *Biogenic amine biosynthesis* and *Peptide hormone processing*.

In order to identify neuropeptide precursors from the EST database, sequences available from different insect species, such as *S. gregaria*, *L. migratoria*, *D. melanogaster*, *A. mellifera* and *A. pisum*, were used in a homology search. Previously identified locust neuropeptides, without known precursor information, were used as well. For this purpose, tblastn [221], adapted for short sequences, was employed. The retrieved results were analyzed using SignalP 3.0 to predict potential signal peptide cleavage sites. Alignments of putative neuropeptide precursors were performed by means of CLUSTALW.

### 4. Comparison to LocustDB

All unique sequences available from the *L. migratoria* EST-database (LocustDB) [25], [26] were used as a query to do a blastn search in the *S. gregaria* EST-database, and *vice versa*. Sequences producing an E-value<1E-10 were considered a hit.

Next, all unique *L. migratoria* sequences were entered in a new ‘Blast2GO’ functional annotation project (*cfr. supra*). The annotation steps were performed by using the same parameters as for the *S. gregaria* sequences. In order to compare functional annotations in both EST databases, the 100 best represented biological processes (i.e. the best represented GO terms classified under the main ontology ‘Biological Process’) were retrieved for both databases.

### 5. Genes differentially regulated in the two phases

#### Selection of candidate differentially regulated genes

Since different inserts were provided with a specific tag during the cDNA synthesis procedure, ESTs could be identified as derived from head ganglia (HG) or ventral nerve cord (VNC), and from isolated- or crowded-reared locusts. In practical terms, this tag identification was only feasible for the 5′ ESTs derived from the second library (where the tag was incorporated at the 5′ end of the insert), as well as for a limited number of sequences (*i.e.* 5′ ESTs including the tag-containing 3′ end) derived from the first library (where the tag was incorporated at the 3′ end of the insert). Therefore, contig sequences composed by at least five separate, tag containing ESTs were evaluated for the presence of an isolated- or crowded-reared specific tag, allowing us to classify them as carrying substantially more (*i.e.* each with a ratio ≥five to one) (i) crowded- or (ii) isolated-reared tags. Based on this initial analysis, a selection of 16 transcripts was made, for which the expression level in both phases was further investigated by qRT-PCR.

In addition to this selection, the transcripts representing the factors ‘secreted protein acidic and rich in cysteine’ (SPARC) (LC.3176.C1.Contig3329) and ‘phase-related peptide’ (PRP) (LC.463.C1.Contig551), which were both previously shown to be upregulated in crowded-reared locusts, and the ‘solitarious phase specific gene’ (SSG) (LC01031A1A08.f1), which was previously found to be upregulated in isolated-reared locusts [186]–[188] were included as (putative) positive controls in the subsequent qRT-PCR analysis.

#### Quantitative real-time reverse transcription PCR

To investigate whether the above selected sequences indeed correspond to phase-dependently regulated genes, their transcript levels were evaluated in locust nervous tissue. Sampling was done from adult males and females staged between 4 and 10 days after ecdysis. HG and VNC from crowded- and isolated-reared animals were microdissected and pooled as separate samples. Each condition was represented by four biological repeats (each containing pooled tissues from 10 animals). RNA extraction was performed as described in the above section. Subsequent cDNA synthesis was done with SuperScript III Reverse Transcriptase (Invitrogen Life Sciences) by using 1 µg of the total RNA and random hexamers as described in the manufacturer's protocol. All primers for these qRT-PCR analyses were designed by means of the Primer Express software (Applied Biosystems) and their sequences are represented in Table 4 and Table 5. QRT-PCR was performed in a 20 µl reaction volume, as described in the Fast SYBR Green Master Mix protocol (Applied Biosystems). The final concentration of the primers was 300 nM. All reactions were run in duplicate on a StepOnePlus Real-Time PCR system (Applied Biosystems), using the following thermal profile: holding stage at 95°C (10 min), followed by 40 cycles of 95°C (3 s) and 60°C (30 s). Then a dissociation protocol was performed allowing for a melt curve analysis. In order to compensate for small differences in reverse transcription efficiency, we also evaluated the transcript levels for two endogenous controls in the samples under study. A random selection of samples covering all conditions and tissues was used to determine the most stable endogenous control genes. The tested genes were the desert locust orthologs of β-actin, elongation factor 1α (EF1α), ribosomal protein Rp49, glyceraldehyde-3-phosphate dehydrogenase, tubulin A1, ubiquitin and CG13220 [225]. EF1α and the *S. gregaria* ortholog for CG13220 were selected by the GeNorm software [226] for having the most stable transcript levels in the studied samples. Relative quantities of transcript levels were calculated as described by Vandesompele *et al.*, 2002 [226]. One of the pooled HG samples derived from crowded-reared locusts was used in every qRT-PCR run as a calibrator cDNA sample. Transcript levels for specific genes in the samples under study were calculated relatively to levels for the same transcript in the calibrator sample. Statistical analysis was performed by means of Microsoft Excel analysis software and consisted of a Student's t-test for comparing two independent groups. A level of p<0.05 was considered as significant.

## Supporting Information

Figure S1 **The 100 best represented GO terms (** ***Biological Process*** **) in the** ***S. gregaria*** **EST database.** Overview of the 100 best represented GO terms classified under the main ontology *Biological Process* in the *S. gregaria* EST database.
(PNG)

Figure S2 **The 100 best represented GO terms (** ***Biological Process*** **) in the** ***L. migratoria*** **EST database.** Overview of the 100 best represented GO terms classified under the main ontology *Biological Process* in the publicly available *L. migratoria* EST database LocustDB [25], [26].
(PNG)

Table S1 **Overview of EST sequences representing neuropeptide precursors.**
(DOC)
