# Supplementary figures and images for: Transcriptome Analysis of the Desert Locust Central Nervous System: Production and Annotation of a Schistocerca gregaria EST Database
Source: PLoS One. 2011 Mar 21;6(3):e17274. doi: 10.1371/journal.pone.0017274 (PMC3061863; doi:10.1371/journal.pone.0017274)

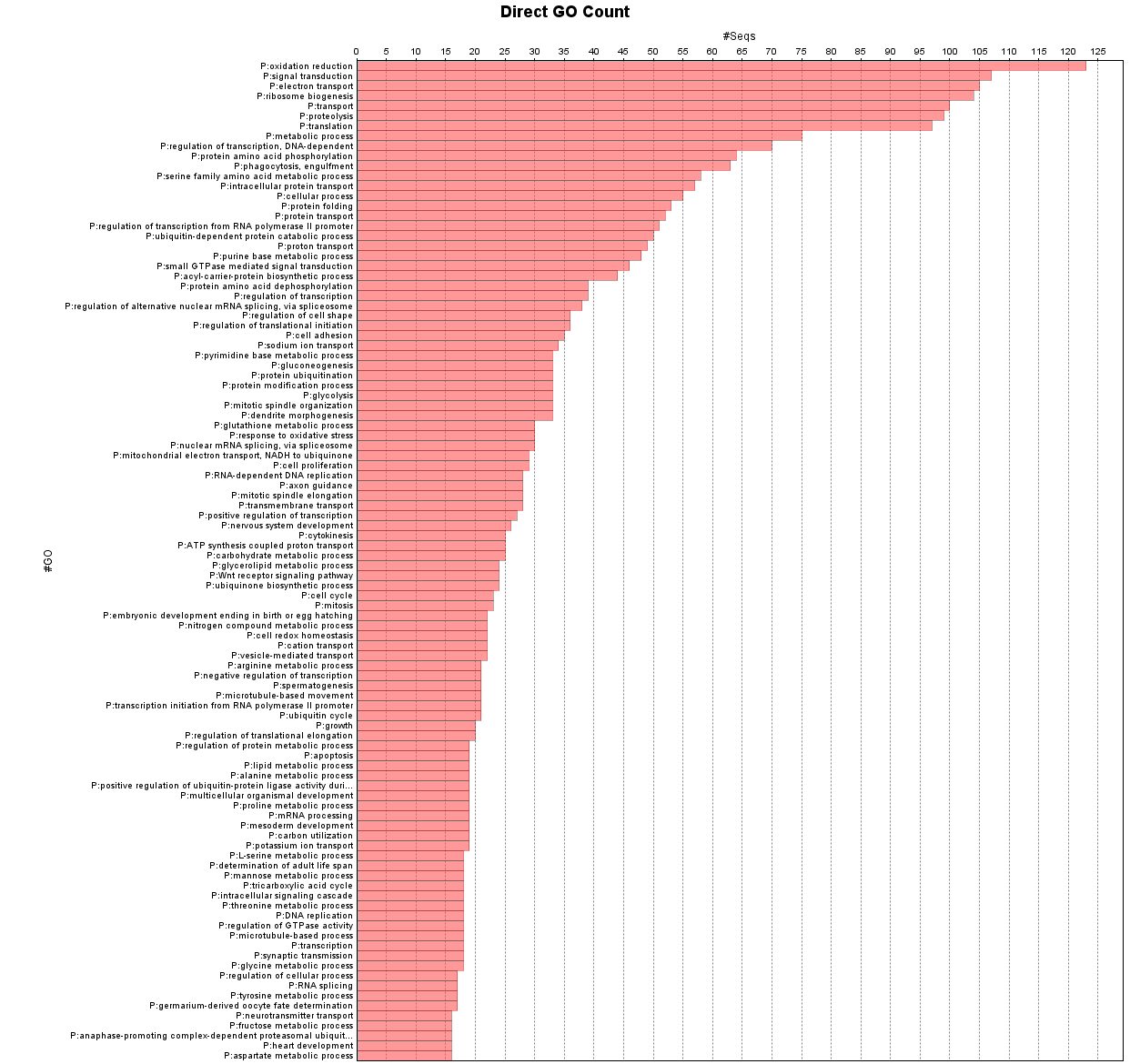

Supplement: Figure S1 — The 100 best represented GO terms ( Biological Process ) in the S. gregaria EST database. Overview of the 100 best represented GO terms classified under the main ontology Biological Process in the S. gregaria EST database. (PNG) [file pone.0017274.s001.png]

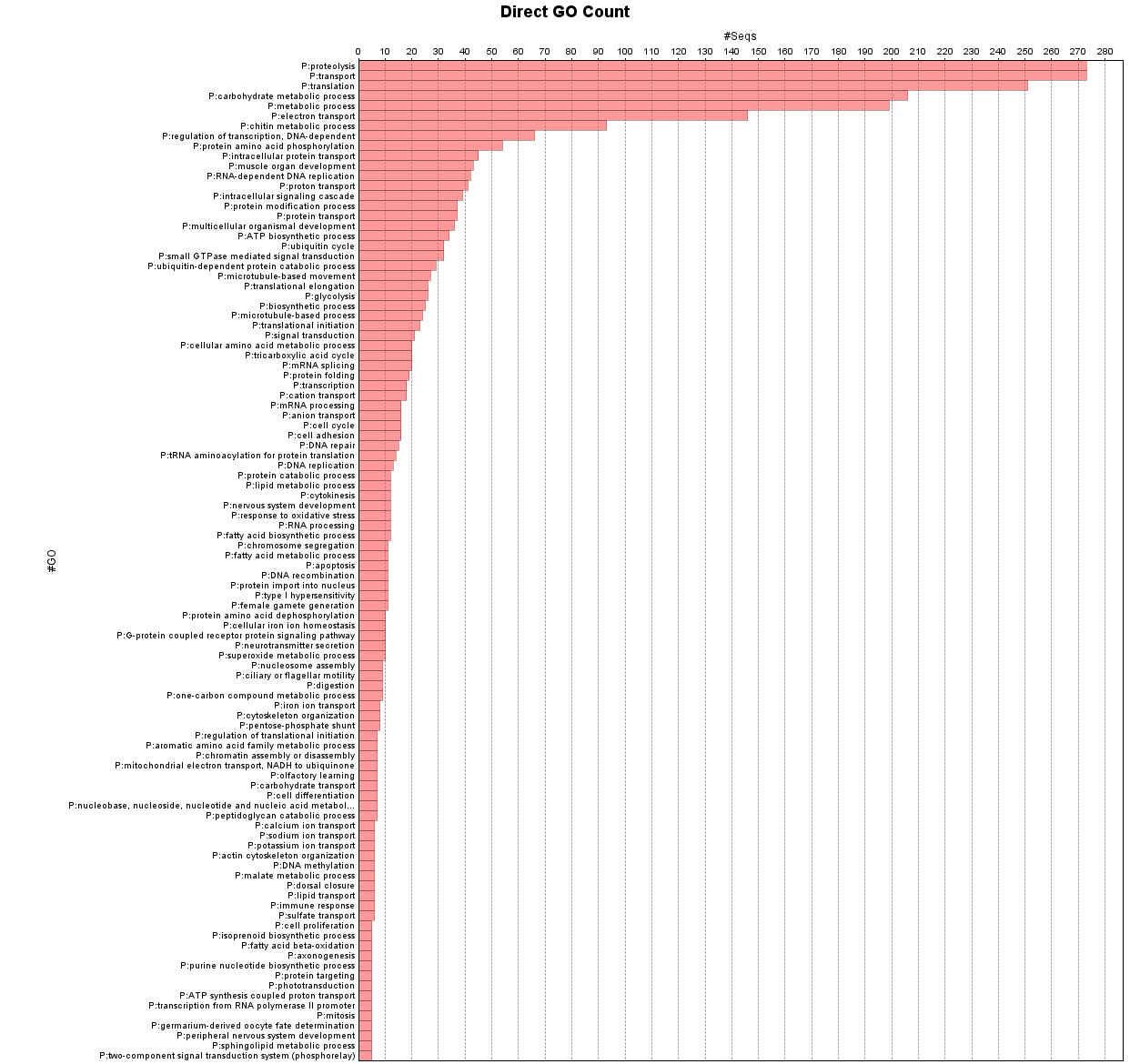

Supplement: Figure S2 — The 100 best represented GO terms ( Biological Process ) in the L. migratoria EST database. Overview of the 100 best represented GO terms classified under the main ontology Biological Process in the publicly available L. migratoria EST database LocustDB [25], [26]. (PNG) [file pone.0017274.s002.png]
