# Supplementary material for: Transcriptome Analysis of the Desert Locust Central Nervous System: Production and Annotation of a Schistocerca gregaria EST Database
Source: PLoS One. 2011 Mar 21;6(3):e17274. doi: 10.1371/journal.pone.0017274 (PMC3061863; doi:10.1371/journal.pone.0017274)
Supplement: Table S1 — Overview of EST sequences representing neuropeptide precursors. (DOC) [file pone.0017274.s003.doc]

**Table S1. Overview of EST sequences representing neuropeptide precursors.**

| **Complete neuropeptide precursors** | | |
| --- | --- | --- |
| **Name** | **Contig nr.** | **Translated Amino Acid Sequence** |
| AKH I | LC.540.C2.Contig639 | *MVQRCLVVALLVVVVAAALCSA***QLNFTPNWGT**g***KR*DAADFGDPYSFL**YRLIQAEARKMSGCSN |
| AKH II | LC.1180.C1.Contig1324 | *MLVVAVCAALSAA***QLNFSTGW**g***RR*YADPNADPMAFL**YKLIQIEARKLSGCSN |
| AT | LC.2005.C1.Contig2167 | *MRCAAAALCLLVALAALCAAAAA*APAAHYGRGSRPRTI***R*GFKNVALSTARGF**g***KR***DGNQLEAALADRDTTLPDSFPVEWFAAEMQNNPELARMIVSKFVDANQDGELTAEELLRPTY |
| AST CC | LC.407.C1.Contig492 | *MSTAVKAVLLLVVALAATCWARA*EPLGQQPSDKARLLNELDLVDDDGSIETALINYLFAKQVVNRLRAQMDVSDLQR***KR*SYWKQCAFNAVSCF**g***K*** |
| CCHa | LC01024B1A06.f1 | *MSAKQIPAQPGCARLPTMALVLALAVTLALLQAADA****KR*GCMAFGHSCFGGH**g***KR***ADMEPAAEGVEGAEEAAAVAEAEAAAAAALLDDAASPQRFRLSPFLRQWLQRAYQQQSADSQTVEVK |
| GPA | LC.3116.C1.Contig3268 | *MVPPSSRSALHFFALAVALCLSAVSA***GMDGERDAWEKPGCHRVGHTRKISIPDCIEFPITTNACRGFCESWSVPSALNTLRVNPHQAITSIGQCCNIMETEDVEVRVMCLDGPRDLVFKSAKSCQCYHCKKD** |
| HrTH | LC.3853.C1.Contig3980 | *MVARLFLALTVTAWCCYLVTS***QVTFSRDWSP**g***KR***SPEPTCAKHAASICQILVNELRQLAACEMKSLLRYHAEEVNVPQEIYIDGNGGR |
| ITP-S | LC01048A2B09.f2 | *MHHQKQQQQQKQQGEAPCRHLQWRLSGVVLCVLVVASLVSTAAS***SPLDPHHLA*KR*SFFDIQCKGVYDKSIFARLDRICEDCYNLFREPQLHSLCRSDCFKSPYFKGCLQALLLIDEEEKFNQMVEIL**g***KK*** |
| ITP-L | LC.1384.C1.Contig1529 | *MHHQKQQQQQKQQGEAPCRHLQWRLSGVVLCVLVVASLVSTAAS***SPLDPHHLA*KR*SFFDIQCKGVYDKSIFARLDRICEDCYNLFREPQLHSLCRKDCFTSDYFKGCIDVLLLQDDMDKIQSWIKQIHGAEPGV** |
| NPP-1 | LC.3439.C1.Contig3577 | *MKPAAALAAATLLIAVILFHRAEA***NPISRSCEGANCVVDLTRCEYGEVTDFFGRKVCAKGPGDKCGGPYELHGKCGDGMDCRCGVCSGCSMQSLECFFFEGAAPNSC** |
| NPF | LC.1768.C1.Contig1921 | *MSQSRPLALLVLSAAVVALLLVVAAPAPAEA***QQAAADGNKLEGLADALKYLQELDRYYSQVARPRF**g***KR***SELRPDVVDDVIPEETSADKFWRRFARRR |
| TK | LC.108.C1.Contig162 | *MCRVGALLLMAALVACEGAAG*QGPDAGEQRGPADAAAFLRMRAAGGDGDGKDALLE***KR*APLLGFHGVR**g***KK***DDLDELD***KR*APSLGFHGVR**g***KK***DDADDDDGFD***KR*APLRGFQGVR**g***KK***DEADAEDAELGDGADYLQLADLPYREDYDADADADADELQLDDPWLRDE***KR*ALKGFFGTR**g***KK*APQAGFYGVR**g***KK*GPSGFYGVR**g***KK*APLSGFYGVR**g***KK*APSLGFHGVR**g***KK***DDGASPPDLDSLLYYLNEASEATRQ***KR*GNT*KK*APVGFYGTR**g***KK***SWAPDGGAASSSDSIAPSLINSQ |
| ITG | LC.817.C1.Contig945 | *MWLIGRLVQVVVVLALLNGGALG*WGGLFNRFSPEMLSNLGYGGHGYGAYRSSQPLLQRFHNPVEVFQELQEDEEPCYGKKCTSNEHCCPGTVCVDVDGIVGSCLFAYGLKQGELCRRDSDCETGLLCADSADGRTCQPPLTNRKQYSEDCTMSSECDISKGLCCQLQRRHRQAPRKVCSYFKDPLICIGPVAADQVKEDIEHTAGE***KR*ITGKVASFNHI*RR***K |
| **Incomplete neuropeptide precursors** | | |
| **Name** | **Contig nr.** | **Translated Amino Acid Sequence** |
| AST A | LC01039B2D01.f2 | …XXX**LYDFGV**g***KR*AYTYVSEY*KR*LPVYNFGL**g***KR*ATGAASLYSFGL**g***KR*GPRTYSFGL**g***KR***GDDEPNDYSEQELFADVDGDSEDALPVAVEADERELPEAAEEEMPGVFTELMD***KR*GRLYSFGL**g***KR*ARPYSFGL**g***KR*AGPAPSRLYSFGL**g***KR*EGRMYSFGL**g… |
| Burs-β | LC01070B1E02.f1 | …VVRAPLEVDGIDKLDIEFRCCRCQWACNSQVQPSVTTPTGFLKECYCCRESFLRERTVTLSHCYDPDGARLTAEGTATMDIRLREPAECKCFKCGDFSR |
| Cap-2b / PVKs | LC.2414.C1.Contig2580 | …KL***KK*TSSLFPHPRI**g***R***SEFINHEAVAEQPSPFEGH***KR*KGLVANARV**g***RR*DGAETPGAAASLWFGPRV**g***R*AGLGQDETRAGTK*RR*GLLAFPRV**g***R***GHAGSSSSSSSGDGDGDGA***R*DSLWFGPRV**g***RR***E***RR*SLRL*R*LPAAAWLAAGDVGNGKGDFTPRL**g***R***ESGEEEATVLLVGDGNTAEGFDAVADADIDEEER |
| CCAP | LC01021B2A10.f1 | …VTSPADAVSRPPGPAGVSSAGRRRRRHHGEEGYGLAVPQSTVEAKM***KR*PFCNAFTGC**g***KK***RSDESVGTLLEMNSEPAVADLSRQILSEAKLWEAIQEARAELLARRRQHEMQTNRLADFSRPLAVAQYRKKRAAAPPTPVQGMKPWRR |
| DH | LC.1479.C1.Contig1625 | …XANXXXGGPPEVDXIDKLDIEFRCCRRCCRERARPTRPSRPARLRTPSRDVRQADPRPARV***KR*MGMGPSXXIVNPMDVLRQRLLLEIARRRLRDAEEQIKANKDFLQQI**g***KR***SPHAGGAANDAGADAPPFGLRAAAERSASDISKDWASSDSRWNNQFTVRQS |
| OMP_PAA_ | LC01029A2F01.f1 | *MSPVRVLVAALLAVSCGGGCSA***YYEAPPDGQRLLLQAAPAAAPAAPAAASWPHQQRRQAIDEFAAA**… |
| OMP | LC01039A2H08.f2 | *MSPVRVLVAALLAVSCGGGCSA***YYEAPPDGQRLLLQAAPAAAPAAASWPHQQRRQAIDEFAAA**… |
| NPP-2 | LC01023B1G08.f1 | **…TGCSMHTLQCYSDFSTPTTCP** |
| NPLP | LC.1768.C1.Contig1921 | ***…KR*YLASLVRSHGLPYPLT*KK*EDDGPGEI*KR*NVGALARNWMLPS**g***KR*ASDDDQEVD*KR*YLASVLRQ**g***R***SDGFRQNSDGAQQADHEEE***KR*HLGSLAKSGMAIH*KK***TSRSAGSDGQAFLQQQQQQEQGGAHAQDAAGS***KR***S***KR***HAYLLPPAPPQSLAPAPGEFPMPVLQNNDDALDYGDLLDLMSDVLGAPE***KR*FLGVPPAAADY**g***KR*HIGALARLGWLPSFRAASA*R*SG*R*SAGSRS**g***KR***ATRSHSADGPWPAELQQA |

The (partial) neuropeptide precursor amino acid sequence is shown (italics: signal peptide, bold: neuropeptide, bold italics: dibasic cleaving site, lowercase ‘g’: glycine amidation signal). Abbreviations: AKH: adipokinetic hormone; AT: allatotropin; AST: allatostatin; GPA: glycoprotein hormone α; HrTH: hypertrehalosemic hormone; ITP-S: short ion transport peptide; ITP-L: long ion transport peptide; NPP: neuroparsin precursor; NPF: neuropeptide F; TK: tachykinin; Burs-β: bursicon β-subunit; Cap-2b: cardioactive peptide; CCAP: crustacean cardioacceleratory peptide; DH: diuretic hormone; OMP: ovary maturating parsin; OMP_PAA_: ovary maturating parsin displaying the PAA insertion; NPLP: neuropeptide-like precursor. The nomenclature of CCHa is based on the fact that this neuropeptide has two conserved cysteine residues and an amidated histidine residue. The nomenclature of Apis ITG is based on a pattern of three amino acid residues in the sequence.
